# Increased canonical NF-kappaB signaling specifically in macrophages is sufficient to limit tumor progression in syngeneic murine models of ovarian cancer

**DOI:** 10.1186/s12885-020-07450-8

**Published:** 2020-10-07

**Authors:** Alyssa A. Hoover, Demetra H. Hufnagel, Whitney Harris, Kennady Bullock, Evan B. Glass, Esther Liu, Whitney Barham, Marta A. Crispens, Dineo Khabele, Todd D. Giorgio, Andrew J. Wilson, Fiona E. Yull

**Affiliations:** 1 Department of Pharmacology, Vanderbilt University, Nashville, TN 37232 USA; 2 Vanderbilt University School of Medicine, Nashville, TN USA; 3 Department of Biomedical Engineering, Vanderbilt University, Nashville, TN USA; 4 Department of Obstetrics and Gynecology, Division of Gynecologic Oncology, Vanderbilt University Medical Center, Nashville, TN USA; 5 Vanderbilt-Ingram Cancer Center, Nashville, TN USA; 6 Department of Obstetrics and Gynecology, Division of Gynecologic Oncology, Washington University School of Medicine, St. Louis, MO USA

**Keywords:** NF-κB, Ovarian cancer, Tumor immunology

## Abstract

**Background:**

New treatment options for ovarian cancer are urgently required. Tumor-associated macrophages (TAMs) are an attractive target for therapy; repolarizing TAMs from M2 (pro-tumor) to M1 (anti-tumor) phenotypes represents an important therapeutic goal. We have previously shown that upregulated NF-kappaB (NF-κB) signaling in macrophages promotes M1 polarization, but effects in the context of ovarian cancer are unknown. Therefore, we aimed to investigate the therapeutic potential of increasing macrophage NF-κB activity in immunocompetent mouse models of ovarian cancer.

**Methods:**

We have generated a transgenic mouse model, termed IKFM, which allows doxycycline-inducible overexpression of a constitutively active form of IKK2 (cIKK2) specifically within macrophages. The IKFM model was used to evaluate effects of increasing macrophage NF-κB activity in syngeneic murine TBR5 and ID8-Luc models of ovarian cancer in two temporal windows: 1) in established tumors, and 2) during tumor implantation and early tumor growth. Tumor weight, ascites volume, ascites supernatant and cells, and solid tumor were collected at sacrifice. Populations of macrophages and T cells within solid tumor and/or ascites were analyzed by immunofluorescent staining and qPCR, and soluble factors in ascitic fluid were analyzed by ELISA. Comparisons of control versus IKFM groups were performed by 2-tailed Mann-Whitney test, and a *P*-value < 0.05 was considered statistically significant.

**Results:**

Increased expression of the cIKK2 transgene in TAMs from IKFM mice was confirmed at the mRNA and protein levels. Tumors from IKFM mice, regardless of the timing of doxycycline (dox) administration, demonstrated greater necrosis and immune infiltration than control tumors. Analysis of IKFM ascites and tumors showed sustained shifts in macrophage populations away from the M2 and towards the anti-tumor M1 phenotype. There were also increased tumor-infiltrating CD3^+^/CD8^+^ T cells in IKFM mice, accompanied by higher levels of CXCL9, a T cell activating factor secreted by macrophages, in IKFM ascitic fluid.

**Conclusions:**

In syngeneic ovarian cancer models, increased canonical NF-κB signaling in macrophages promoted anti-tumor TAM phenotypes and increased cytotoxic T cell infiltration, which was sufficient to limit tumor progression. This may present a novel translational approach for ovarian cancer treatment, with the potential to increase responses to T cell-directed therapy in future studies.

## Background

Ovarian cancer is the 5th leading cause of cancer deaths among U.S. women [1]. Patients are often diagnosed at late-stage, due to non-specific early symptoms and lack of effective early detection methods; with the majority of patients diagnosed with advanced disease, 5-year relative survival is less than 50% [1]. Many cases are initially responsive to first line platinum-based chemotherapeutics, but will ultimately experience chemoresistant disease recurrence [2]. New therapeutic approaches are urgently required to treat these patients and support tumor surveillance.

The therapeutic potential of targeting cells in the tumor microenvironment (TME) that support tumor growth and metastasis is being increasingly recognized. Overcoming T cell exhaustion by tumor cells via checkpoint inhibitor therapy has led to durable responses in some solid tumor types, but ovarian cancer patients have shown relatively poor response rates at least in part due to low numbers of tumor-infiltrating T cells [3]. Tumor-associated macrophages (TAMs) are highly enriched in the TME and have emerged as an attractive alternative target for therapy. High numbers of tumor-infiltrating macrophages are an indicator of poor prognosis in a variety of solid tumors, including ovarian cancer [4–7]. However, the ability to exploit these large macrophage populations in patients who have poor responses to traditional treatments and adaptive immunotherapies could provide a new direction in cancer therapeutics [8–11].

Macrophages are highly plastic and can be classified on a polarization spectrum, ranging from anti-tumor M1 to pro-tumor M2 phenotypes [12]. TAMs are predominantly M2 macrophages that respond to anti-inflammatory cytokines, contribute to immunosuppressive functions, and promote angiogenesis to sustain tumor growth [4, 6, 13]. In contrast, M1 macrophages are activated by exposure to pro-inflammatory cytokines and microbial products, produce immunostimulatory products, and mediate T cell responses to pathogens [14]. M2 macrophages have been characterized by expression of markers such as the mannose receptor (CD206), early growth response protein 2 (Egr2), arginase-1 (Arg-1), and interleukin-10, while M1 macrophages are characterized by expression of markers such as inducible nitric oxide synthase (iNOS), CD38, tumor necrosis factor alpha (TNFα), C-C motif chemokine ligand 3 (CCL3), and major histocompatibility complex class II (MHCII) [14, 15]. While much of the focus in macrophage cancer immunotherapy has been on the elimination of TAMs in the TME, recent efforts have focused on harnessing the high numbers of TAMs in the TME for therapeutic benefit by re-education into an M1, anti-tumor phenotype to overcome immunosuppression [16–18].

Accumulating evidence from our group and others indicates that a key regulator of macrophage behavior is canonical nuclear factor-kappaB (NF-κB) signaling, via nuclear translocation and binding of p65/p50 transcription factors [18–24]. Although one study found that inhibition of canonical NF-κB signaling in TAMs leads to the anti-tumorigenic M1 phenotype [22], an increasing body of literature demonstrates the opposite, finding that increased NF-κB activity in macrophages polarizes them towards an M1-like anti-tumorigenic phenotype. We have shown that knockdown of cytoplasmic IκBα, inhibitor of NF-κB, promotes a shift towards the M1 phenotype and induces cytotoxicity towards tumor cells in cultured macrophages [23, 24]. Moreover, complementary in vivo studies in transgenic mice overexpressing a constitutively activated form of cytoplasmic IKK2 specifically in macrophages, termed IKFM, show reduced growth and metastatic seeding of injected melanoma and mammary tumor cells [19, 21].

Often diagnosed at advanced stage, ovarian cancer is commonly characterized by extensive metastatic seeding of tumor cells throughout the peritoneal cavity [1]. Moreover, macrophages account for over 50% of the total cell population in ascites and peritoneal implants in epithelial ovarian cancer [8]. Therefore, we hypothesized that activation of NF-κB in macrophages in mice injected with ovarian cancer cells would induce a shift towards the M1 phenotype in TAMs and limit tumor progression. To test this hypothesis, we combined our IKFM transgenic mice with established syngeneic TBR5 or ID8-Luc models of ovarian cancer [25–29]. We showed that there was therapeutic benefit in ovarian tumor-bearing IKFM mice compared to controls, accompanied by an M1 phenotype shift in TAMs and increased infiltration of CD3^+^/CD8^+^ T cells. Our data provide evidence that high TAM numbers in the context of ovarian cancer can be exploited by a therapeutic strategy that modifies their behavior towards anti-tumor phenotypes. The potential of our approach to improve responses to T cell-directed therapy is also highlighted, which will be the focus of future studies.

## Methods

### IKFM transgenic mouse model

All animal experiments were approved by the Vanderbilt University Institutional Animal Care and Use Committee (IACUC) and were conducted in accordance with American Association for Laboratory Animal Science (AALAS) and ARRIVE guidelines. All transgenic mice were on an FVB or C57BL/6 strain background and were generated by our group and produced by breeding from our existing colony [19]. Effects of targeted activation of NF-κB in macrophages on ovarian tumorigenesis were examined using an established double transgenic mouse model, termed IKFM [19]. To generate IKFM double transgenics, mice harboring the reverse tetracycline transactivator (*rtTA*) gene under the control of the monocyte/macrophage-specific colony stimulating factor receptor 1 promoter, c*fms*-*rtTA* (FMR), were crossed with mice harboring the NF-κB activating (tet-O)7-FLAG-cIKK2 transgene (IKK). In IKFM mice treated with doxycycline (dox), rtTA protein binds to the tet operon, driving transcription of FLAG-cIKK2 in macrophages. Littermates lacking one or both transgenes were used as controls. Mouse genotypes were established by PCR as follows: ear clips were digested overnight at 37 °C in 500 μL tail digest buffer (0.3 M Sodium Acetate, 1 M TrisHCL pH 7.9, 0.5 M EDTA pH 8.0, and 10% Sodium Dodecyl Sulfate) and 5 μL of Proteinase K. The following day, samples were homogenized prior to use in PCR analysis. To identify the presence of cIKK2 transgenic DNA, samples were combined with the primer pair (FOR 5′-CTT CTC ATG ATC TGG ATC TCC 3′, REV 5′-GAC GCC ATC CAC GCT GTT TTG 3′) and GoTaq Flexi DNA polymerase (Promega, M8295) to yield a product size of 452 bp. For the FMR transgene, the primer pair (FOR 5′- CCA TGT CTA GAC TGG ACA AGA-3′, REV 5′- CTC CAG GCC ACA TAT GAT TAG-3′) was used with the RedTaq ReadyMix PCR Reaction (Sigma, R2523) to yield a 550 bp product. All mice included in these studies were females aged 12–24 weeks of age who had not previously been injected with tumor cells or treated with dox, and who exhibited normal behavior and appearance on visual examination prior to tumor cell injection. Mice were group housed (maximum 5 mice/cage) with standard bedding and ad libitum access to food and water. Animal rooms were maintained at constant temperature and humidity with a 12-h light/dark cycle. Mice were assigned to experimental groups based on genotypes (control versus IKFM), and were randomized such that each experimental cage receiving dox water contained a mix of control and IKFM mice. All experiments were completed with *n* = 5 mice in each treatment group and each experiment was independently replicated twice. Based on power calculations using the Vanderbilt Power and Sample Size Program, we determined that a total of 10 mice/experimental subgroup was sufficient for 80% power to detect changes of 22% between group means at a standard deviation of 15% and a *P*-value significance threshold of 0.05 [30].

### Cell culture and tumor injection

Mouse ovarian cancer cell lines used were BRCA-mutated (p53^−/−^, Brca1^−/−^, myc) TBR5 cells in experiments with FVB mice, and ID8 cells expressing a constitutive luciferase reporter plasmid (ID8-Luc) for C57BL/6 experiments [25–29]. Both cell lines were cultured in 10% FBS-supplemented DMEM High-Glucose medium with 1% penicillin/streptomycin using standard techniques. Prior to injection, TBR5 or ID8-Luc cells were passaged to 80–90% confluence. Cells were counted, resuspended in sterile PBS at a concentration of 25 million cells/ml, and 5 million cells were injected intraperitoneally (IP) with a sterile 3 mL LuerLok syringe and 21G needle.

### Doxycycline treatment of IKFM and control mice

IKFM and control female mice injected intraperitoneally (IP) with tumor cells were kept on regular drinking water until transgene activation was desired. At the appropriate timepoint (defined below), both IKFM and littermate controls were treated with freshly prepared water containing 1 g/L doxycycline (dox; Sigma Aldrich, St. Louis, MO) and 5% sucrose given *ab libitum*. The dox water was stored in a red bottle to prevent light-induced dox degradation and was replenished twice per week. Dox stimulation to drive expression of cIKK2 in macrophages was given at different time intervals to model two clinically relevant states: 1) established tumors: in FVB mice injected with TBR5 cells, dox treatment was started at 7 days post-injection, and continued for 14 days before sacrifice except for a 2-day treatment break from days 12–14. For mice on the C57BL/6 background injected with ID8-Luc cells, because of the slower progression of tumorigenesis than TBR5, dox was given from 30 to 60 days post-injection on a metronomic treatment schedule (5 days on, 2 days off) to evaluate effects on established disease, with sacrifice at 60 days post-injection; and 2) implantation and early tumor growth: in FVB mice, dox was started 3 days prior to TBR5 cell injection and continued for 7 days post-injection. Mice were then given normal drinking water for an additional 2 weeks before sacrifice.

### Mice and tumor collection

Prior to sacrifice at the times indicated or when pre-defined humane endpoint criteria approved by the Vanderbilt IACUC were reached, tumor progression was monitored by body weight, with representative data provided in Additional file 1, and mice were checked for signs of cachexia. All mice were euthanized by carbon dioxide with secondary cervical dislocation according to IACUC-approved protocols. Following sacrifice, omental tumor implants were harvested, weighed, and snap-frozen or fixed in 10% neutral-buffered formalin for 48 h for H&E and immunostaining as described below. Ascites was collected by withdrawing fluid from the peritoneal cavity with a hypodermic syringe, and the volume was measured. If no measurable ascites was present, peritoneal lavage was performed by injecting 10 ml PBS IP and carefully extracting the fluid with a hypodermic syringe. Ascites or peritoneal lavages were centrifuged at 1500 rpm for 5 min. The supernatant (soluble fraction) was frozen for analysis of peptide factors as described below. For the cellular component of ascitic fluid or peritoneal lavages, where applicable, red blood cells were lysed with 5 mL Geyz lysing buffer (4.15 g NH_4_Cl, 0.5 g KHCO_3_ in 500 mL MilliQ Water) for five minutes in a 37 °C water bath. The lysis reaction was neutralized with 25 mL cold PBS prior to centrifugation, repeating lysis if necessary. The final cell pellet was suspended in PBS for total cell counts using a Neubauer Improved hemocytometer. A portion of cells were then snap-frozen for RNA extraction or protein analysis as described below.

### Analysis of soluble cytokines in ascites

In the soluble fraction of ascites harvested from FVB IKFM and control mice treated with dox from 1 to 3 weeks after TBR5 injection, the concentration of vascular endothelial growth factor (VEGF) and C-X-C motif chemokine ligand 9 (CXCL9) was measured using a mouse VEGF ELISA kit and mouse MIG/CXCL9 ELISA kit (both from RayBiotech, Norcross, GE, USA) according to manufacturer’s instructions. The optical density at 450 nm was measured using a microplate reader (Tecan Infinite M1000 Pro; Switzerland). For each sample, VEGF or CXCL9 levels were normalized to corresponding total protein levels measured by Bradford protein assay.

### RNA extraction and quantitative RT-PCR (qRT-PCR)

Total RNA from ascites cells was extracted using Trizol (Invitrogen) and Direct-zol RNA Miniprep (Zymo Research). RNA concentration was measured via a NanoDrop 2000 spectrophotometer (Biotek, Winooski, VT, USA). cDNA was synthesized from the RNA via RT-PCR with Maloney-Murine Leukemia Virus Reverse Transcriptase (M-MLV RT, Promega). For detection of the FLAG-tagged cIKK2 transgene, qRT-PCR was utilized on TBR5 ovarian ascites samples (58 °C annealing temperature and 35 cycle program). For all other gene targets, qRT-PCR analysis was run on the CFX96 Touch™ Real-Time PCR Detection System (BioRad) using the PowerUP SYBR Green Master Mix (Applied Biosystems). Primer sequences for the epithelial marker CK18, the pan murine macrophage marker F4/80, the M1 macrophage markers (CCL3, iNOS, TNFα, and CD38) and the M2 macrophage markers, (Egr2, IL-10, and mannose receptor) are shown in Additional file 2. Each primer pair was tested and melt curves analyzed to ensure that only a single amplicon was generated. All experimental and control samples were assayed in triplicate for target or reference genes. For all experiments, two reference genes were analyzed (GAPDH and B2M). Similar results were obtained using either reference gene, and values shown are relative to corresponding B2M or GAPDH expression levels using the 2-^ΔΔCt^ comparative method, as specified [31].

### Immunofluorescence analysis

Processing, embedding and sectioning of formalin-fixed tumor tissue, and hematoxylin and eosin staining for histology, were performed by the Translational Pathology Shared Resource at Vanderbilt [32]. Immunofluorescent staining of harvested tumor tissue and mounting of slides was performed as previously described [33]. The following primary antibodies were used: rat polyclonal anti-F4/80 (Cat# MCA497, AbD Serotec, 1:200), rabbit polyclonal anti-arginase-1 (Cat# GTX109242, GeneTex, 1:200), mouse monoclonal anti-FLAG tag H-5 (Cat# sc-166,355, Santa Cruz, 1:100), mouse-anti mouse CD3 (Cat# sc-1179, Santa Cruz, 1:500), and rat anti-mouse CD8 (Cat# NBP1-49045SS, Novus Biologicals, 1:100). Secondary antibodies (all used at 1:1000) were goat anti-rat Alexa Fluor 488 (Cat# ab150157, Abcam), goat anti-mouse Alexa Fluor Cy5 (Cat# ab6563, Abcam), and goat anti-rabbit Alexa Fluor 594 (Cat# ab150080, Abcam).

### Imaging and semi-automated image processing and quantification

H&E and colorimetric stained tissues were imaged using the EVOS XL Core microscope (ThermoFisher Scientific) on 10x and 20x brightfield magnification. All fluorescently-labeled tissue slides were imaged on the EVOS FL Auto 2 and EVOS M7000 Imaging Systems. Images were taken on 20X APO and 40X APO including DAPI, Texas Red, Cy5 and GFP channels at a resolution of 300 pixels per inch. For immunofluorescence quantification, 5 fields with at least 200 cells were randomly selected from each slide to be counted. On solid tumor sections, 3 of these fields were taken from the edge of the tumor, and 2 fields were obtained from internal tumor to account for variability in the distribution of immune cell tumor infiltration. To reduce observer bias, semi-automated analysis of immunofluorescence was conducted using the R package *EBImage*. Images in each fluorescent channel were blurred and thresholded using adaptive thresholding, to account for changes in underlying background signal caused by uneven illumination or by stray signal from nearby bright objects. Segmentation was performed using connected set labeling and after manual review of thresholded images, parameters were set such that non-cellular objects with small and large surface areas were removed. Objects in each channel were then counted and counts from the DAPI channel were used to define the number of total nucleated cells in a field. All microscopy figures were generated using Adobe Photoshop CC 2018 (Version 19.0.1).

### Western blot

We performed western blot analysis of FLAG expression and NF-κB signaling in cell pellets harvested from ascites from control and IKFM ID8-Luc-injected C57BL/6 mice. Subcellular fractionation, western blotting, and signal detection were performed as previously described [34]. Primary antibodies used were rabbit polyclonal anti-FLAG (Abcam; 1:100) and rabbit polyclonal anti-p65 (Cell Signaling Technology; 1:1000). We confirmed the efficiency of fractionation using mouse monoclonal anti-histone H3 (Cell Signaling Technology; nuclear specific marker; 1:1000 dilution) and mouse monoclonal anti-α-tubulin (Sigma Chemical Co.; cytoplasmic specific marker; 1:1000 dilution). All original, uncropped gels are available in Additional file 3.

### Statistical analysis and diagrams

Statistical analyses were performed and corresponding figures were generated using GraphPad Prism (Version 8: La Jolla, California, USA). We generally preferred a non-parametric approach given small sample sizes. Comparisons of control versus IKFM groups in in vivo experiments were performed by 2-tailed Mann-Whitney test. A *P*-value < 0.05 was considered statistically significant. Data are generally plotted graphically as mean vertical bars with standard error of the mean. Additional diagrams were created by the authors in Microsoft PowerPoint 2016 (Version 16.16.26) and with BioRender.com.

## Results

### Doxycycline-inducible transgenic mouse model system to modulate NF-κB signaling in macrophages in syngeneic ovarian cancer models

**Fig. 1:**
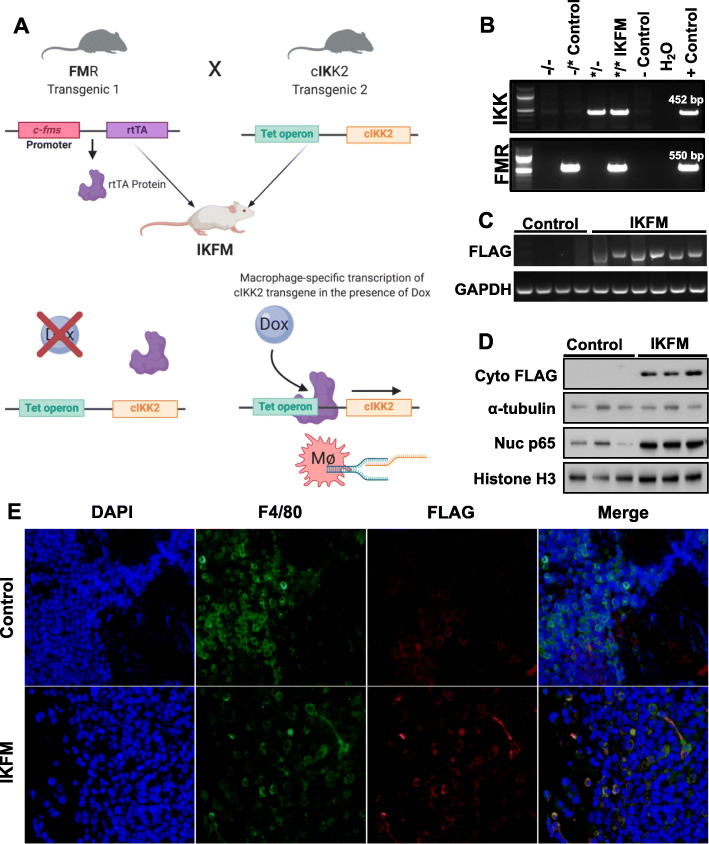
The doxycycline-inducible IKFM model of NF-κB activation in macrophages. **a** Schematic of generation of IKFM double transgenics. Mice harboring an rtTA transgene gene under the control of a macrophage-specific *c-fms* promoter (FMR) are crossed with mice harboring a FLAG-cIKK2 transgene under the control of a doxycycline-inducible rtTA-responsive Tet operon (IKK). **b** Representative DNA genotyping gels for double transgenic IKFM mice (FLAG-cIKK2 and c-fms-rtTA positive) mice and control mice (c-fms-rtTA positive only). IKFM and control mice injected with tumor cells were treated with 1 g/L dox using schedules for treating established TBR5 and ID8-Luc tumors as defined in Methods. FLAG-cIKK2 transgene expression was confirmed in IKFM macrophages by **c** qRT**-**PCR analysis of mRNA expression from TBR5 ascites cellular fraction in FVB IKFM mice. Equal loading was confirmed by corresponding GAPDH levels. **d** Western blot analysis of cytoplasmic FLAG and nuclear p65 protein levels in macrophages isolated from ID8-Luc ascitic fluid from C57Bl/6 IKFM mice. Equal loading was confirmed by probing for cytoplasmic α-tubulin and nuclear histone H3. Brightness was reduced uniformly across the gel image to increase visualization of faint bands. **e** Representative images from immunofluorescence analysis of TBR5 omental tumor tissue from FVB IKFM mice showing tumor-infiltrating macrophages; nuclei (blue); F4/80 (green); FLAG-IKK2 (red). Figure 1**a** was created by the authors with BioRender.com and Fig. 1**b**-**e** were generated with Microsoft PowerPoint 2016 (Version 16.16.26) and Adobe Photoshop CC 2018 (Version 19.0.1)

Our team has developed several unique transgenic mouse models to study the role of NF-κB signaling during tumorigenesis in specific cell types and at defined temporal intervals [19, 35, 36]. One such model, termed IKFM, allows doxycycline-inducible expression of a constitutively activated form of FLAG-tagged IKK2 (FLAG-cIKK2) to upregulate canonical NF-κB signaling in macrophages [19]. To create the IKFM model, (tet-O)7-FLAG-cIKK2 (IKK) transgenics were mated with colony stimulating factor receptor 1 promoter-reverse tetracycline transactivator *c-fms*-*rtTA* (FMR) transgenics to produce IKK/FMR double positive mice. In IKFM but not control mice, dox treatment allows binding of the rtTA protein to the tet operon, driving transcription of the FLAG-cIKK2 transgene (Fig. 1a). Identification of IKFM mice and control littermates lacking one or both transgenes is performed by PCR genotyping (Fig. 1b).

We have previously shown that dox treatment in IKFM mice results in decreased metastatic seeding and tumor growth of melanoma and mammary cancer cells [19, 21]. Since advanced ovarian cancer is a disease characterized by dissemination of tumors throughout the peritoneal cavity, we hypothesized that NF-κB activation in ovarian tumor-associated macrophages would also limit tumorigenesis in well-defined syngeneic ovarian cancer mouse models. Murine ovarian cancer cells, TBR5 and a luciferized ID8-derivative (ID8-Luc), were introduced into the peritoneal cavity of control or IKFM mice, where they implanted to generate solid omental tumor masses along with accumulation of ascites [26, 37]. In tumor-bearing control and IKFM FVB mice treated with dox, we confirmed specific mRNA expression of the FLAG-cIKK2 transgene in IKFM macrophages isolated from ascitic fluid (Fig. 1c). We also performed western blot analysis of ascites cells from ID8-Luc-injected mice to show selective FLAG-cIKK2 expression at the protein level (Fig. 1d). Moreover, FLAG-cIKK2 expression in macrophages was associated with increased levels of nuclear p65, indicative of active signaling, in IKFM mice compared to controls (Fig. 1d). Finally, we also confirmed selective protein expression of FLAG-cIKK2 in harvested tumors, since F4/80-positive tumor-infiltrating macrophages co-stained with FLAG in IKFM mice, but not controls, in immunofluorescence analysis (Fig. 1e).

### In established ovarian tumors, activation of NF-κB in macrophages alters tumor morphology and reduces the percentage of M2 macrophages in tumors and ascitic fluid

**Fig. 2:**
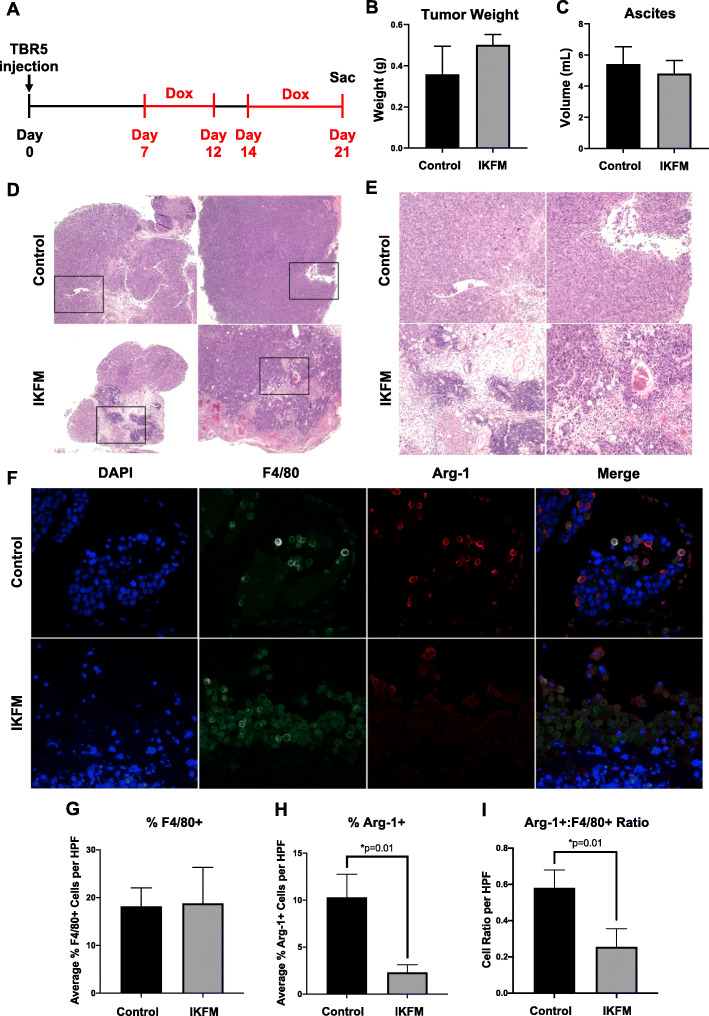
Effects of NF-κB activation in established TBR5 tumors in IKFM mice. FVB IKFM and control mice injected with TBR5 cells were treated with 1 g/L dox from 7 to 21 days post-tumor cell injection (in red), with a two-day break from days 12–14. **a** Schematic of experimental design. **b** Harvested omental tumor weight and **c** ascites volume at sacrifice. Values are mean + SEM; *n* = 10 per group. **d** Representative low-power 4x magnification H&E images of each group, with **e** showing corresponding high-power 20x images of the boxed areas. **f** Representative images of immunofluorescent staining of harvested TBR5 omental tumors for F4/80 (macrophages; green), arginase-1 (M2 macrophage marker; Arg-1; red), and DAPI (nuclei; blue). Semi-automated counts of **g** percent of F4/80 positive cells per high-power field (HPF), **h** percent of Arg-1 positive cells per HPF, and **i** the ratio of Arg-1^+^ to F4/80^+^ cells. Counts were quantified from 5 high-power fields from each representative mouse sample and represent percentage of cell subtype relative to total cellularity in each field. Values are mean + SEM of *n* = 3 for each group; *P*-values are from the Mann-Whitney test. Figure 2**a** was generated with Microsoft PowerPoint 2016 (Version 16.16.26); Fig. 2**b**, **c**, and **g-i** were generated with GraphPad Prism (Version 8: La Jolla, California, USA); and Fig. 2**f** was generated with Adobe Photoshop CC 2018 (Version 19.0.1)

Ovarian cancer most commonly presents as advanced, metastatic disease [1]. Therefore, we first tested dox dosing schedules to model treatment of established disease, with short periods of no treatment to limit possible toxicity associated with systemic inflammation. No adverse effects due to dox administration were observed in any experiment described herein. In FVB background mice injected with TBR5 cells, dox treatment began 7 days after injection of TBR5 cells and was continued for 14 days before mouse sacrifice, with a 2-day gap in treatment on days 12–14 post-injection (Fig. 2a). In a second syngeneic model, C57BL/6 background mice were injected with ID8-Luc cells and dox treatment was started 30 days post-injection and continued on a metronomic (5 days on, 2 days off) schedule for 30 days before mouse sacrifice (Additional file 4A). There were no significant changes in weight of harvested omental tumors or volume of ascites between control and IKFM mice injected with TBR5 cells (Fig. 2b & c). However, clear differences in the histology of harvested tumors were observed. These included numerous areas of focal necrosis and inflammatory infiltration in tumors from IKFM mice compared to controls, suggestive of increased, localized anti-tumor activity (Fig. 2d & e). Similarly, we observed greater tumor heterogeneity and extensive inflammatory infiltration in IKFM tumors compared to controls in the ID8-Luc syngeneic model, but no overall change in tumor burden measured by ascites volume and harvested tumor weight (Additional file 4B-D).

Previous studies from our group have shown that increasing canonical NF-κB activity in macrophages is associated with a shift away from the M2 pro-tumor phenotype [23, 24]. We therefore used immunofluorescence analysis to examine whether the anti-tumor effects observed in IKFM mice were accompanied by reduced M2 polarization of TAMs. Semi-automated counts of cells positive for the pan macrophage marker F4/80 and the murine M2 macrophage marker arginase-1 (Arg-1) were performed as described in Methods. In harvested omental tumors from IKFM mice injected with TBR5 cells, there was no overall increase in infiltrating F4/80^+^ macrophages compared to controls (Fig. 2f & g). However, a significantly decreased number of tumor-infiltrating macrophages positive for Arg-1 was observed in IKFM mice (Fig. 2f & h). Importantly, there was also a significant reduction in the ratio of F4/80 positive macrophages co-staining with Arg-1 in IKFM tumors, indicating a shift away from the pro-tumorigenic M2 phenotype in IKFM TAMs (Fig. 2i).

**Fig. 3:**
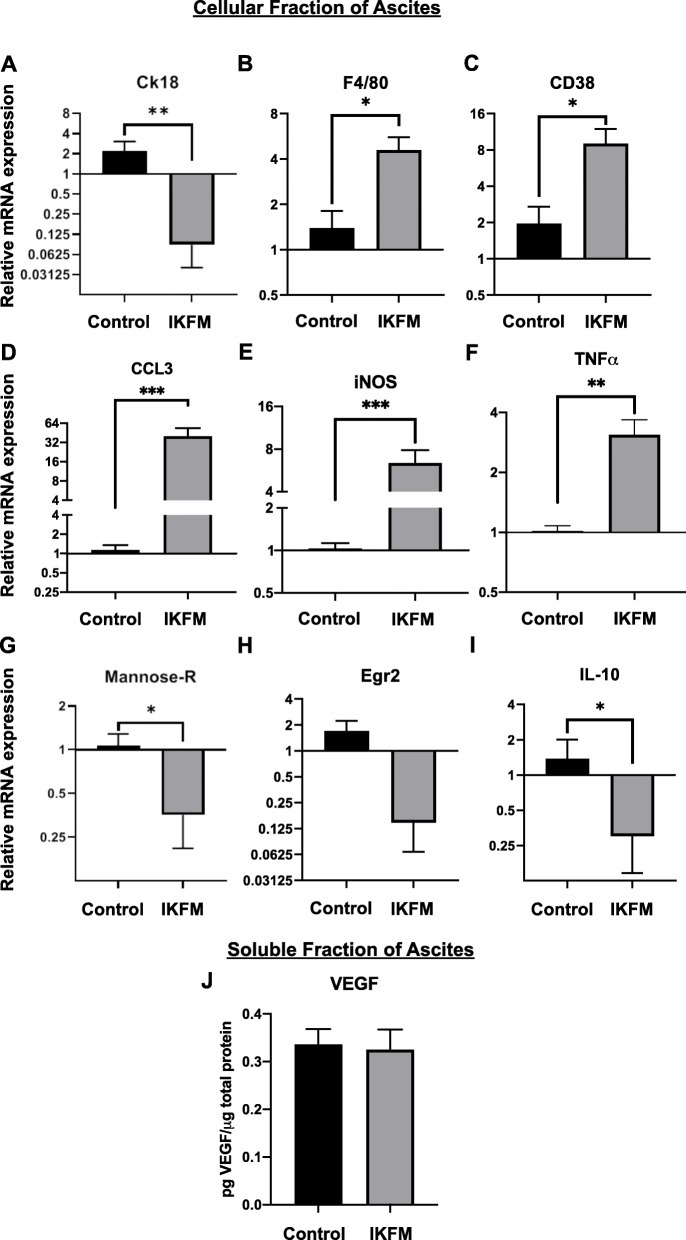
Shift from M2 towards M1 phenotypes in ascites cells from IKFM mice. FVB IKFM and control mice injected with TBR5 cells were treated with 1 g/L Dox from 7 to 21 days post-tumor cell injection, with a two-day break from days 12–14. In harvested ascites cells, mRNA levels of the following were measured by qRT-PCR analysis: **a** epithelial tumor marker CK18, **b** pan macrophage marker F4/80, **c-f** M1 macrophage markers (CD38, CCL3, iNOS, and TNFα), **g-i** M2 macrophage markers (mannose-receptor, Egr2 and IL-10). Values were calculated using the 2^-ΔΔCt^ method relative to corresponding levels of the B2M **a-c**, **g-i** or GAPDH **d-f** internal control. Values are shown in log2 scale and are mean + SEM (*p < 0.05, **p < 0.005, ***p < 0.001 relative to control, Mann-Whitney test). **j** VEGF levels in the soluble fraction of ascites from FVB IKFM mice were measured by ELISA. Values were expressed relative to corresponding protein content and represent mean + SEM for each group measured in duplicate (*n* = 3 control, *n* = 4 IKFM). Figure 3**a**-**j** were generated with GraphPad Prism (Version 8: La Jolla, California, USA)

We also examined effects of increased NF-κB signaling on macrophage phenotype in a second niche of the tumor microenvironment, ascitic fluid, which contains predominantly macrophages and floating tumor cells. In cells harvested from ascites from IKFM mice, there was significantly decreased expression of the epithelial tumor cell marker CK18 (Fig. 3a), and significantly increased expression of the F4/80 macrophage marker (Fig. 3b). Consistent with reduced M2 macrophage polarization observed in IKFM solid tumors, ascites from IKFM mice showed significantly increased expression of the M1 macrophage markers CD38, CCL3, iNOS, and TNFα (Fig. 3c-f), and reduced expression of M2 markers, mannose receptor (CD206), Egr2, and the M2-associated cytokine IL-10 (Fig. 3g-i). Collectively, these data suggest a reduction of tumor cells in the ascites of IKFM mice with concurrent increase in total macrophages polarized towards an anti-tumor M1 phenotype.

Since vascular endothelial growth factor (VEGF) is a pro-tumor angiogenic factor known to be produced by M2 macrophages, we measured VEGF levels in the soluble fraction of ascitic fluid [37]. No significant differences in VEGF levels between control and IKFM mice were observed (Fig. 3j), which suggests that the anti-tumor effects in IKFM mice were not mediated through anti-angiogenic mechanisms.

### Upregulation of NF-κB in macrophages during tumor implantation results in reduced tumor burden and persistent macrophage M1 polarization

**Fig. 4:**
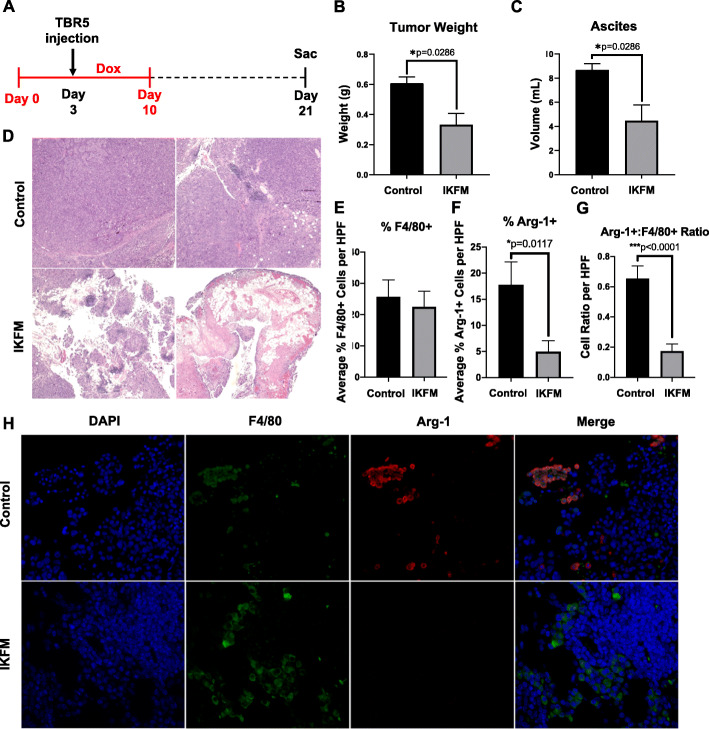
Decreased tumor burden and M2 macrophages following NF-κB activation in macrophages during tumor implantation. FVB IKFM and control mice were treated with 1 g/L dox from 3 days before TBR5 cell injection until 7 days post-injection (in red). Mice were sacrificed 14 days after being taken off dox. **a** Schematic of experimental design. **b** Harvested omental tumor weight and **c** ascites volume at sacrifice. Values are mean + SEM of *n* = 10 per group. **d** Representative low power 4x magnification H&E images of control and IKFM tumors. Semi-automated counts of **e** percent of F4/80 positive cells per high-powered field (HPF), **f** percent of Arg-1 positive cells per HPF, and **g** the ratio of Arg-1^+^ to F4/80^+^ cells. Counts were quantified from 5 high power fields from each representative sample and represent percentage of cell subtype relative to total cellularity in each field. **h** Representative images of tumors stained for F4/80 (macrophages; green), arginase-1 (M2 macrophage marker; Arg-1; red), and DAPI (nuclei; blue). Values are mean + SEM of *n* = 3 for each group; *P*-values are from the Mann-Whitney test. Figure 4**a** was generated with Microsoft PowerPoint 2016 (Version 16.16.26); Fig. 4**b**, **c**, and **e**-**g** were generated with GraphPad Prism (Version 8: La Jolla, California, USA); and Fig. 4**d** and **h** were generated with Adobe Photoshop CC 2018 (Version 19.0.1)

Our studies above showed that induction of cIKK2 in tumor-associated macrophages in already established tumors resulted in localized tumor necrosis accompanied by induced M1 macrophage polarization. A second timeline of dox treatment was used to model a different aspect of ovarian tumorigenesis, implantation and early growth of disseminated tumors, which is relevant to tumor recurrence following debulking surgery and/or primary treatment. In FVB mice, dox treatment was started 3 days prior to TBR5 cell injection and continued for 7 days post-injection; dox was then withdrawn for an additional 2 weeks prior to sacrifice (Fig. 4a). Even more pronounced anti-tumor effects were observed in this model compared to established tumors. Significant decreases in ascites volume and weight of harvested omental tumors were observed in IKFM mice in this treatment window (Fig. 4b & c). There were also striking differences in tumor morphology between control and IKFM tumors. Compared to the relatively homogenous tumors in control mice, IKFM tumors had large areas of necrosis throughout the tumor mass and extensive inflammatory infiltrates (Fig. 4d).

Similar to our macrophage phenotype results from the established tumor model, there was a prominent shift away from the M2 phenotype in tumor-infiltrating macrophages (Fig. 4e-h). Notably, these analyses were performed on tumors harvested 2 weeks after cessation of dox stimulation of FLAG-cIKK2 expression; thus, the shift towards an anti-tumor macrophage phenotype by increased NF-κB signaling was both robust and persistent in our ovarian cancer model.

### Increased infiltration of CD3^+^CD8^+^ T cells into solid tumors after upregulation of NF-κB in macrophages

**Fig. 5:**
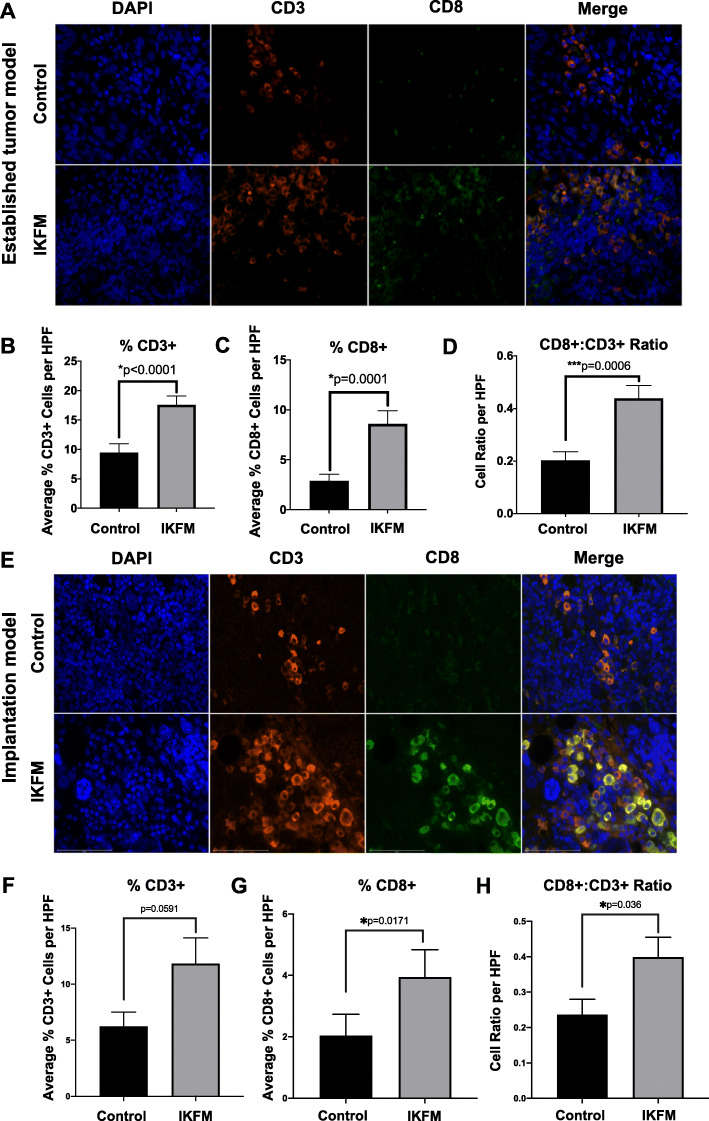
Increased cytotoxic T cell infiltration in IKFM tumors in established tumor and implantation models. Harvested TBR5 omental tumors from the established tumor model (**a-d**) and implantation model (**e-h**) were stained for the pan T cell marker CD3 (red) and the cytotoxic T cell marker CD8 (green) for immunofluorescence analysis. DAPI-stained nuclei are in blue. **a**, **e**) Representative 40x immunofluorescent images. Semi-automated counts of total CD3 positive cells (**b** & **f**) and total CD8 positive cells (**c** & **g**) were performed, with the ratio of CD8 to CD3 positive cells shown in **d** & **h**. Counts were quantified from 5 high-power fields and represent percentage of cell subtype relative to total cellularity in each field. Values are mean + SEM of *n* = 3 for each group; *P*-values are from the Mann-Whitney test. Figure 5**a** and **e** were generated with Adobe Photoshop CC 2018 (Version 19.0.1). Figure 5**b**-**d** and **f**-**h** were generated with GraphPad Prism (Version 8: La Jolla, California, USA)

We next examined effects on T cell infiltration into tumors for two reasons. First, M2 TAMs are known to induce immunosuppressive effects, especially limiting cytotoxic T cell responses [38]. Moreover, our histological studies in both treatment schedules indicated a prominent inflammatory infiltrate in IKFM tumors, consistent with increased anti-tumor immunity. In harvested tumors, we stained sections for the pan T cell marker CD3 and the cytotoxic T cell marker CD8 by immunofluorescence and performed semi-automated analysis to quantify positively staining cells. There were significantly increased overall percentages of CD3 and CD8 positive tumor-infiltrating T cells in the established TBR5 tumor model, and an approximately 2-fold increase in the CD8^+^/CD3^+^ ratio, in IKFM tumors compared to controls (Fig. 5a-d). Similarly, we also observed a trend towards increased T cell infiltration in IKFM tumors in the established ID8-Luc model, which just failed to reach statistical significance (*p* = 0.057, Mann-Whitney test), as shown in Additional file 5. Finally, similar significant increases in cytotoxic T cells were obtained in the tumor implantation and early growth TBR5 model at sacrifice 2 weeks after exogenous NF-κB stimulation, again demonstrating that the increases in anti-tumor immunity in IKFM mice were persistent (Fig. 5e-h).

### Upregulation of T cell recruitment factor CXCL9 in ascites from IKFM mice

To provide insight into the mechanism resulting in the increased recruitment of cytotoxic T cells into the tumor microenvironment in IKFM mice, we measured expression of CXCL9 in the soluble phase of ascites. CXCL9 has recently been identified as a critical mediator of anti-tumor T cell responses, and high CXCL9 levels are associated with increased numbers of CD8 positive T cells in ascites and better prognosis in ovarian cancer patients [39, 40].

CXCL9 protein was undetectable in ascites from all assayed control mice while the mean concentration of CXCL9 in the IKFM group was markedly higher at 458.1 + 162.3 pg/ml, or approximately 0.08 + 0.03 pg CXCL9 per microgram of total protein.

**Fig. 6:**
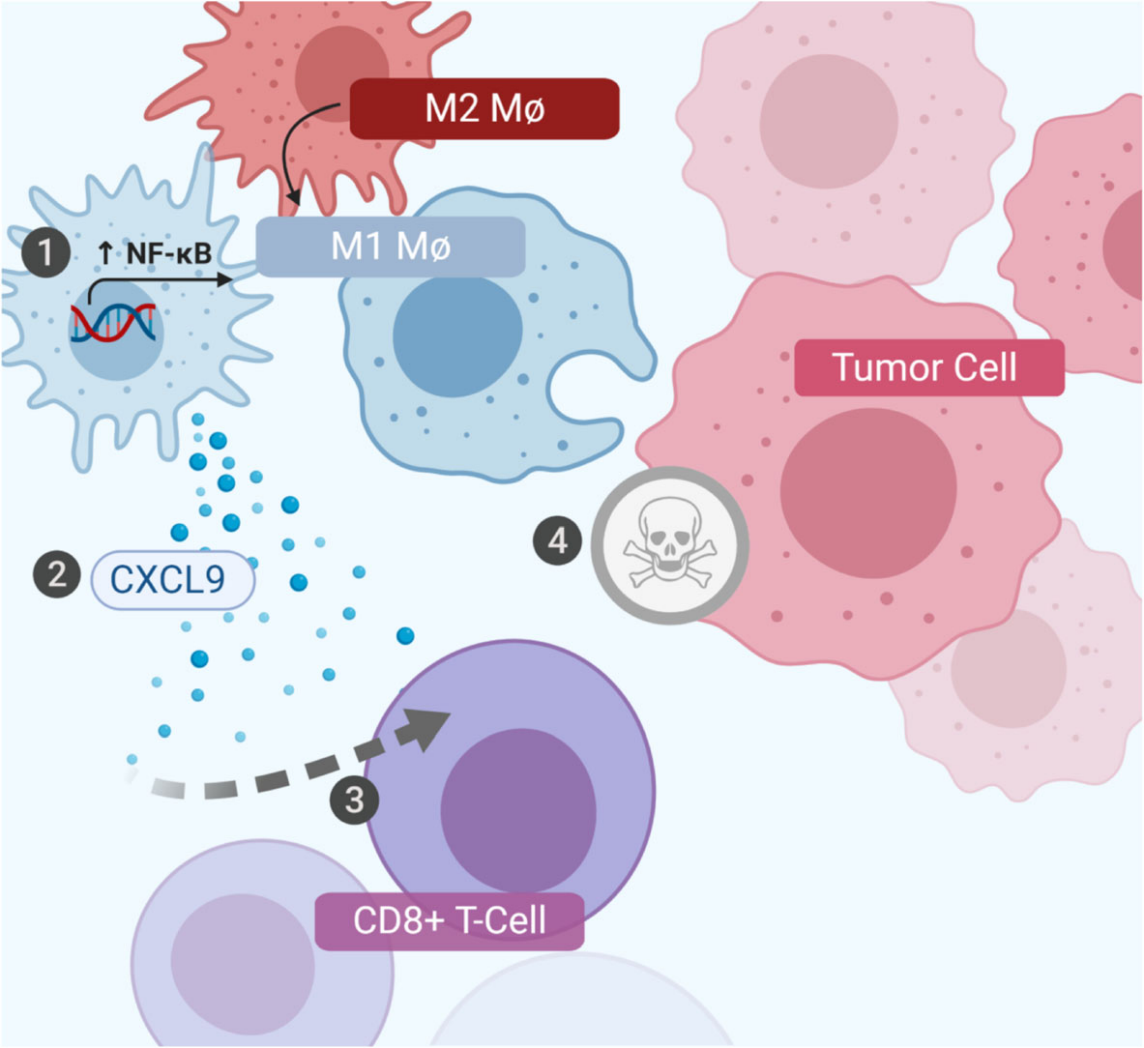
Proposed model of anti-tumor immunity induced by NF-κB activation in tumor-associated macrophages. Activation of NF-κB signaling in tumor-associated macrophages induces polarization of macrophages away from the M2 and towards the anti-tumor M1 phenotype, and also leads to recruitment of cytotoxic T cells, mediated at least in part through increased levels of CXCL9. Figure 6 was created by the authors with BioRender.com

Collectively, our results suggest that increased NF-κB signaling in TAMs induces anti-tumor immunity, including polarization of macrophages away from the pro-tumor M2 phenotype and subsequent recruitment of cytotoxic T cells, with concomitant increases in levels of CXCL9 (Fig. 6).

## Discussion

Interest in the ability to re-educate TAMs is increasing in the field of cancer immunotherapy. Signaling via the NF-κB pathway has been recognized as a critical regulator in the behavior of macrophages in the TME [18–22]. Using the dox-inducible IKFM transgenic model, we previously reported that increased canonical NF-κB signaling in macrophages is sufficient to inhibit metastasis to the lungs of breast cancer or melanoma cells in the context of a tail vein injection model [19, 21]. As there is a critical need for new options for the treatment of ovarian cancer and because epithelial ovarian cancer is characterized by high levels of TAM infiltration, we used the same powerful IKFM model as a tool to gain insights into the effects of upregulating canonical NF-κB signaling in macrophages during ovarian cancer progression. We examined effects in solid tumor implants and ascitic fluid containing non-adherent tumor cells and predominantly macrophages, since they are separate but related tumor microenvironments in ovarian cancer patients. The use of mice was critical in recapitulating the characteristics of human disease. Dox treatment schedules were designed to minimize pain and distress to the mice by reducing the chance of systemic inflammation due to chronic activation of NF-κB signaling. Finally, the experimental group size chosen was the minimum to produce statistically significant data at the desired power level, given the variance observed.

Our first studies were motivated by a rationale to treat existing tumors as would be seen in patients in a clinical setting; treatment with dox began at a clinically-relevant timepoint in the context of already established tumors. Expression of FLAG-IKK2 in TAMs harvested from IKFM but not control mice was confirmed at the mRNA and protein levels, with increased nuclear levels of p65, indicative of active canonical signaling. Initial analyses when animals were sacrificed at end point showed no major differences in tumor weight or ascites volume. However, histological examination of the tumor tissue demonstrated dramatic differences between the control and IKFM groups, including large areas of necrosis and increased immune infiltrate in IKFM tumors. In the clinical setting, such a phenomenon may be seen after initiation of immune-checkpoint inhibitor therapy termed “pseudoprogression” of tumors: as treatment-activated immune cells infiltrate tumors, contributing to necrosis and edema, lesions may transiently increase in volume or remain stable prior to tumor shrinkage [41]. Accordingly, tumor burden, particularly in the setting of immune-modulating treatment, is likely not fully captured by tumor weight, as clinically important immune infiltration and tumor-cell death may be present without resulting in decreased weight. These results were recapitulated with a different syngeneic ovarian tumor cell line (ID8-Luc) in C57Bl/6 mice, indicating a consistent phenotype across different models. Analysis of solid tumor tissue and the cellular component of ascites demonstrated a significant shift away from pro-tumorigenic M2 toward anti-tumorigenic M1 phenotypes among ovarian TAMs in IKFM mice. This is consistent with a previous study showing M1 polarization of TAMs in mammary lung metastases in IKFM mice [19].

We also investigated if similar effects could be seen in the tumor implantation treatment schedule, akin to re-establishment of disease after debulking or chemotherapy. Our findings were similar to the post-implantation model, with the exception that significantly reduced tumor burden and ascites volume was also observed. Given that these benefits were gained even following the cessation of treatment, these data further support the rationale of increasing NF-κB signaling in macrophages to induce persistent shifts towards an anti-tumor phenotype in solid tumor and ascites.

Recent literature has shown a relationship between anti-tumor M1 macrophages and cytotoxic T cell immunity, and much data has shown the importance of CD8^+^ T cells in mediating anti-tumor effects [40, 42, 43]. T cell therapies are exhibiting success in clinical usage but are highly dependent on the target tumor containing a population of infiltrating T cells and, in addition, have a number of auto-immune side effects [44]. While epithelial ovarian cancer is characterized by a predominantly myeloid-derived immune infiltrate, our data from both the pre- and post-implantation models show a shift towards M1 macrophages with concomitant increases in T cells, namely CD8^+^ T cells, together indicating a strong anti-tumor shift in immune populations. Our finding of increased cytotoxic CD8^+^ T cells resulting from increased NF-κB signaling in macrophages presents opportunities to exploit this influx in the tumor microenvironment in a translational setting. CD8^+^ cells and macrophages can work synergistically to exert anti-tumor mediated effects, supporting a rationale for possible combination immunotherapy treatments, such as modulation of macrophage phenotypes together with checkpoint inhibitors to combat T cell exhaustion [45].

Anti-tumor therapy typically targets one or more of three broad anti-tumor mechanisms: overcoming immunosuppression via immune cell stimulation, disruption of angiogenesis, and direct tumor cell killing by conventional chemotherapy and radiation [46, 47]. Since no significant differences in VEGF were detected in ascites, and a prominent influx of both innate and adaptive anti-tumor immune cells into the tumor microenvironment was observed, the IKFM model likely exerts anti-tumor effects through an immune-cell mediated mechanism. These effects may be mediated through a combination of direct tumor cell killing by M1 macrophages, as suggested by unpublished observations from our group, and indirect mechanisms, such as recruitment of cytotoxic lymphocytes.

Recent literature has indicated an important role of the chemokine CXCL9 on the tumor microenvironment, which led us to examine the potential role of this protein in our model [45, 48]. Our finding of significantly upregulated CXCL9 in ascites supernatant has several implications. CXCL9 is a known chemoattractant produced by M1 macrophages that recruits CD8^+^ T cells, and promotes extravasation of leukocytes into the TME, providing a potential mechanism by which we observed increases in cytotoxic T cells following M1 polarization in macrophages [40]. A direct link between NF-κB signaling and CXCL9 downstream production has been reported, suggesting that this effect could be attributed to the increased canonical signaling within macrophages [49]. Critically for the clinical context, recent studies have indicated better prognosis among high-grade serous ovarian cancers with high CD8^+^ T cell infiltrates and CXCL9 levels, supporting the potential clinical significance of our approach [40, 48].

Increasing activity of the canonical NF-κB pathway specifically in macrophages during defined periods of ovarian cancer progression induces anti-tumor immunological changes in the tumor microenvironment. The mechanism by which this occurs includes polarization of macrophages to a more M1-like state, release of anti-tumor, pro-inflammatory cytokines such as CXCL9, and a resultant influx of cytotoxic T cells to promote a “hot” tumor microenvironment. Taken together, the evidence obtained using these transgenic models provides support for the translational potential of this approach for treatment of ovarian cancer. For several years, our team has been developing a nanoparticle-delivery approach to enable the modulation of NF-κB signaling specifically in M2 polarized TAMs in a translational clinical setting for ovarian cancer via targeted siRNA delivery [23]. Moving forward, we hope to translate the findings from these studies to approaches that could be integrated into clinical use, potentially alongside other immunotherapies that require influx of T cells for therapeutic benefit.

## Conclusions

NF-κB signaling is an important mediator of macrophage function and can be manipulated to shift TAMs to anti-tumor phenotypes. In a syngeneic murine model of ovarian cancer, activation of canonical NF-κB signaling specifically in macrophages was sufficient to induce anti-tumor effects, including modulation of macrophages to anti-tumor phenotypes, production of the pro-inflammatory cytokine CXCL9, and infiltration of cytotoxic T cells. This may present a robust translational approach for the treatment of ovarian cancer, and also highlights its potential to increase responses to T cell-directed therapy such as immune checkpoint inhibitors in future studies.

## Supplementary information

**Additional file 1.** Representative body weight data for monitoring of disease progression. Representative body weight data for monitoring of disease progression over the experimental period.

**Additional file 2.** Primer Sequences. All primer sequences referenced in this work.

**Additional file 3.** Original, uncropped gels and blots. All original, uncropped gels and blots included in this study.

**Additional file 4.** Histological differences between IKFM and control tumors in established ID8-Luc tumors. C57BL/6 IKFM and control mice injected with ID8-Luc cells were treated with metronomic 1 g/L dox (5 days on, 2 days off per week) over a period of 30–60 days post-tumor cell injection (in red). **A)** Schematic of experimental design. **B)** Harvested omental tumor weight and **C)** Ascites volume at sacrifice. Values are mean + SEM. **D)** Representative low-power 4x magnification H&E images of control and IKFM tumors, with high-power 20x images of the boxed areas.

**Additional file 5.** Increased T cell infiltration in established ID8-Luc tumors in IKFM mice. C57BL/6 IKFM and control mice injected with ID8-Luc cells were treated with metronomic 1 g/L dox (5 days on, 2 days off per week) over a period of 30–60 days post-tumor cell injection (in red). **A)** Quantification of percent of CD3^+^ T cells via immunohistochemistry (IHC) using a CD3 pan-T cell marker. **B)** Representative high-power CD3 IHC images. Values are mean + SEM (*p* = 0.0571, Mann-Whitney test).

## Data Availability

All datasets generated and analyzed during this study will be made available after garnering institutional approval and enacting appropriate data sharing agreements.

## Abbreviations

Arg-1: Arginase-1
CCL3: C-C Motif Chemokine Ligand 3
CXCL9: C-X-C Motif Chemokine Ligand 9
DMEM: Dulbecco’s Modified Eagle Media
ELISA: Enzyme-Linked Immunosorbent Assay
Erg2: Early Growth Response Protein 2
FBS: Fetal Bovine Serum
IkB: Inhibitor of KappaB
IKK: Inhibitor of KappaB Kinase
IL-10: Interleukin-10
iNOS: Inducible Nitric Oxide Synthase
IP: Intraperitoneal
MHCII: Major Histocompatibility Complex Class II
NF-kB: Nuclear Factor-KappaB
PBS: Phosphate Buffered Saline
qRT-PCR: Quantitative Reverse-Transcription Polymerase Chain Reaction
RT-PCR: Reverse-Transcription Polymerase Chain Reaction
TAM: Tumor-Associated Macrophage
TME: Tumor Microenvironment
TNFα: Tumor Necrosis Factor Alpha
VEGF: Vascular Endothelial Growth Factor
