# Supplementary figures and images for: Increased canonical NF-kappaB signaling specifically in macrophages is sufficient to limit tumor progression in syngeneic murine models of ovarian cancer
Source: BMC Cancer. 2020 Oct 7;20:970. doi: 10.1186/s12885-020-07450-8 (PMC7542116; doi:10.1186/s12885-020-07450-8)

## Slide 1
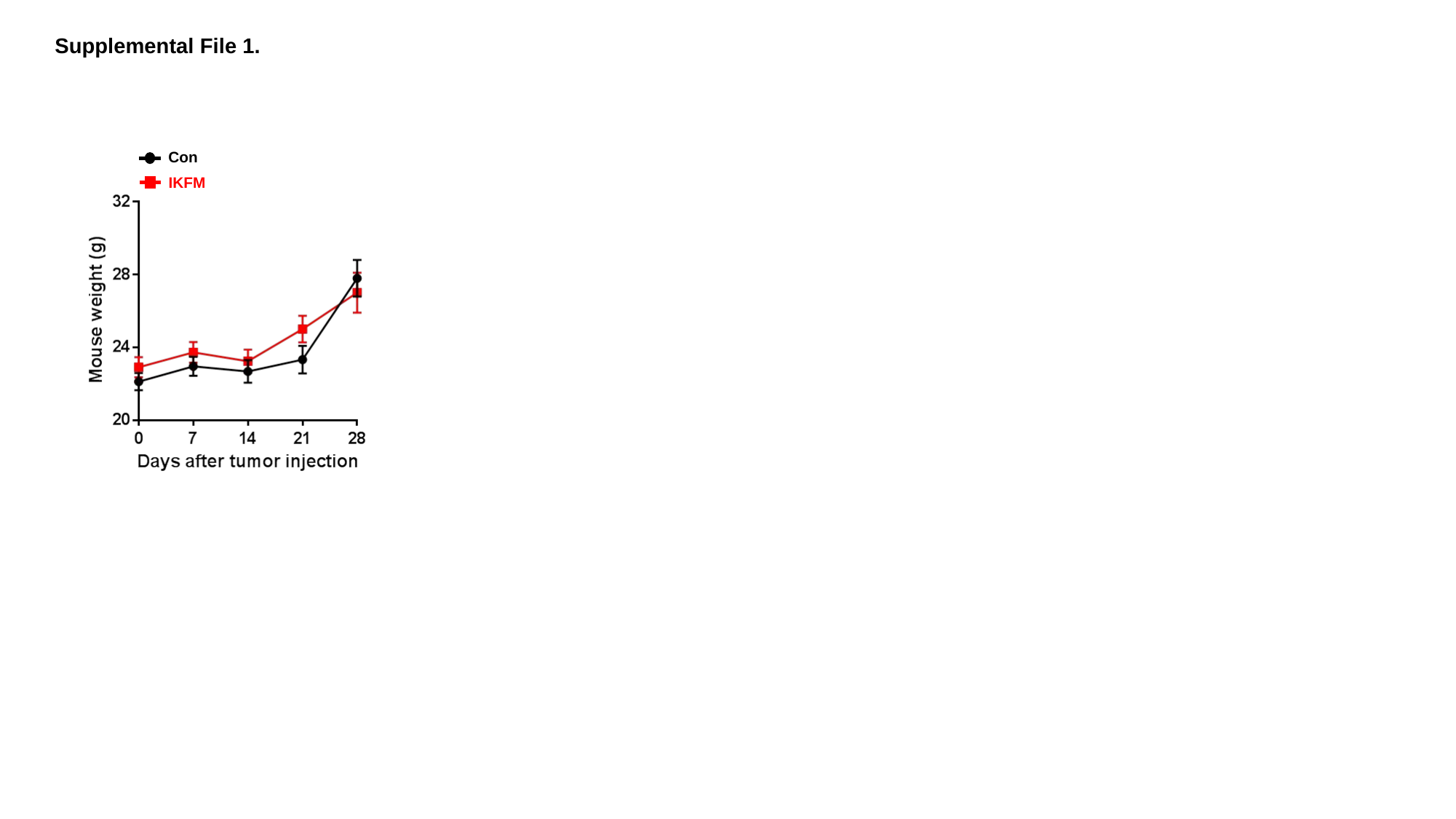

Supplemental File 1.
Con
IKFM

Supplement: Supplementary file 1 — Additional file 1. Representative body weight data for monitoring of disease progression. Representative body weight data for monitoring of disease progression over the experimental period. [file 12885_2020_7450_MOESM1_ESM.pptx]

## Slide 1
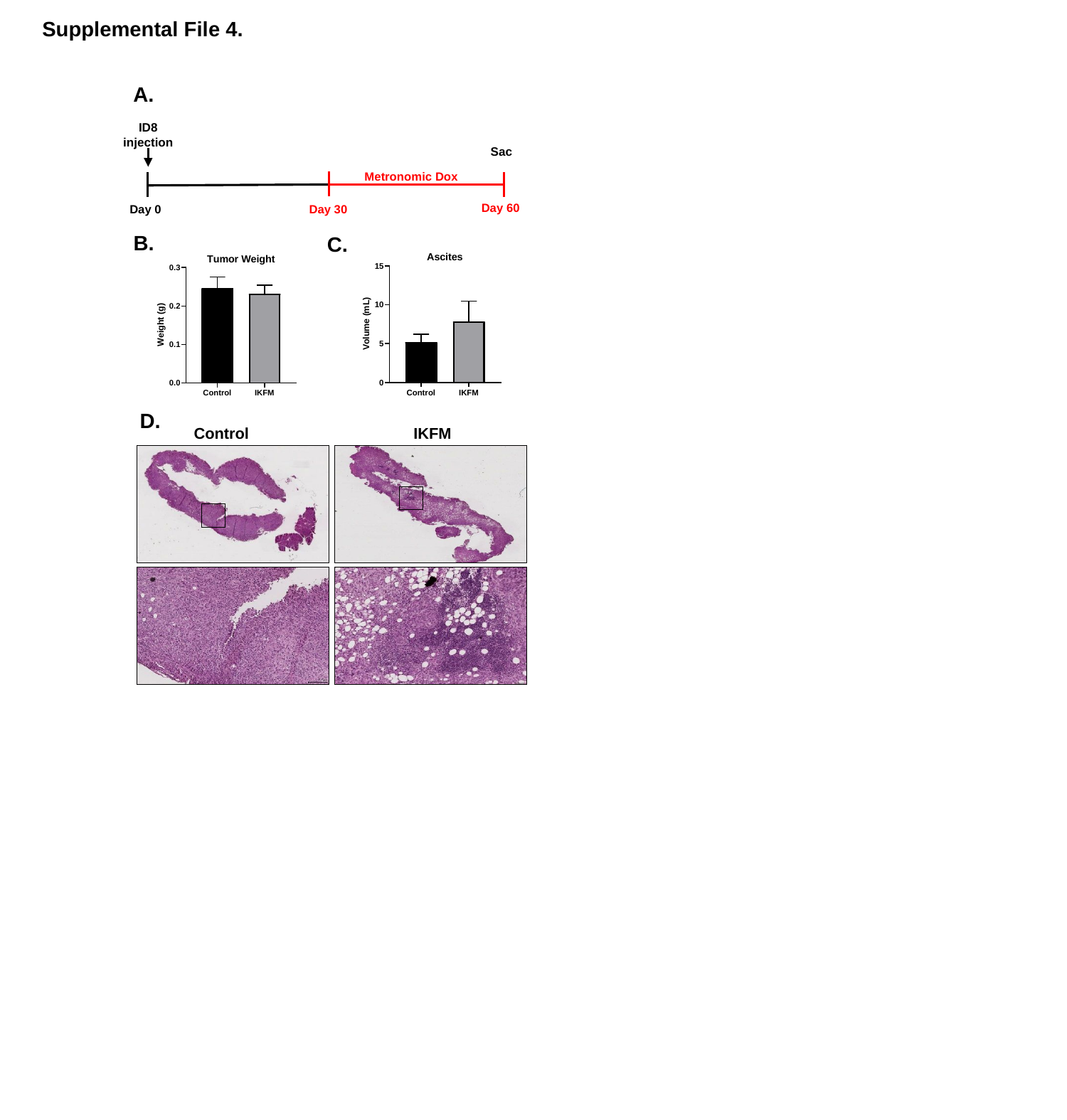

Supplemental File 4.
A.
ID8
injection
Sac
Metronomic Dox
Day 60
Day 30
Day 0
B.
C.
D.
Control
IKFM

Supplement: Supplementary file 4 — Additional file 4. Histological differences between IKFM and control tumors in established ID8-Luc tumors. C57BL/6 IKFM and control mice injected with ID8-Luc cells were treated with metronomic 1 g/L dox (5 days on, 2 days off per week) over a period of 30–60 days post-tumor cell injection (in red). A) Schematic of experimental design. B) Harvested omental tumor weight and C) Ascites volume at sacrifice. Values are mean + SEM. D) Representative low-power 4x magnification H&E images of control and IKFM tumors, with high-power 20x images of the boxed areas. [file 12885_2020_7450_MOESM4_ESM.pptx]

## Slide 1
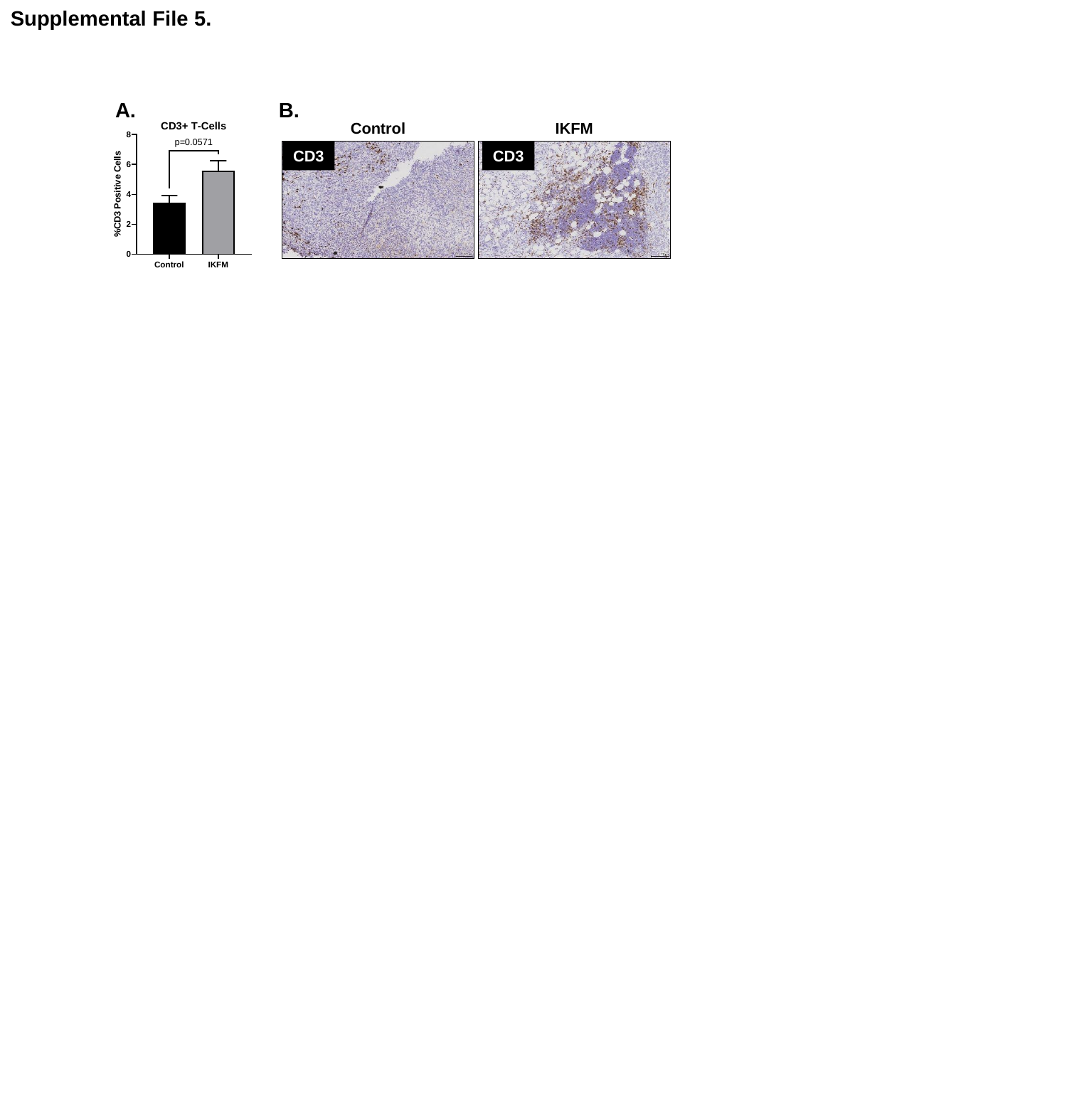

Supplemental File 5.
A.
B.
Control
IKFM
CD3
CD3

Supplement: Supplementary file 5 — Additional file 5. Increased T cell infiltration in established ID8-Luc tumors in IKFM mice. C57BL/6 IKFM and control mice injected with ID8-Luc cells were treated with metronomic 1 g/L dox (5 days on, 2 days off per week) over a period of 30–60 days post-tumor cell injection (in red). A) Quantification of percent of CD3+ T cells via immunohistochemistry (IHC) using a CD3 pan-T cell marker. B) Representative high-power CD3 IHC images. Values are mean + SEM (p = 0.0571, Mann-Whitney test). [file 12885_2020_7450_MOESM5_ESM.pptx]
