# Supplementary material for: Increased canonical NF-kappaB signaling specifically in macrophages is sufficient to limit tumor progression in syngeneic murine models of ovarian cancer
Source: BMC Cancer. 2020 Oct 7;20:970. doi: 10.1186/s12885-020-07450-8 (PMC7542116; doi:10.1186/s12885-020-07450-8)
Supplement: Supplementary file 3 — Additional file 3. Original, uncropped gels and blots. All original, uncropped gels and blots included in this study. [file 12885_2020_7450_MOESM3_ESM.pptx]

## Slide 1
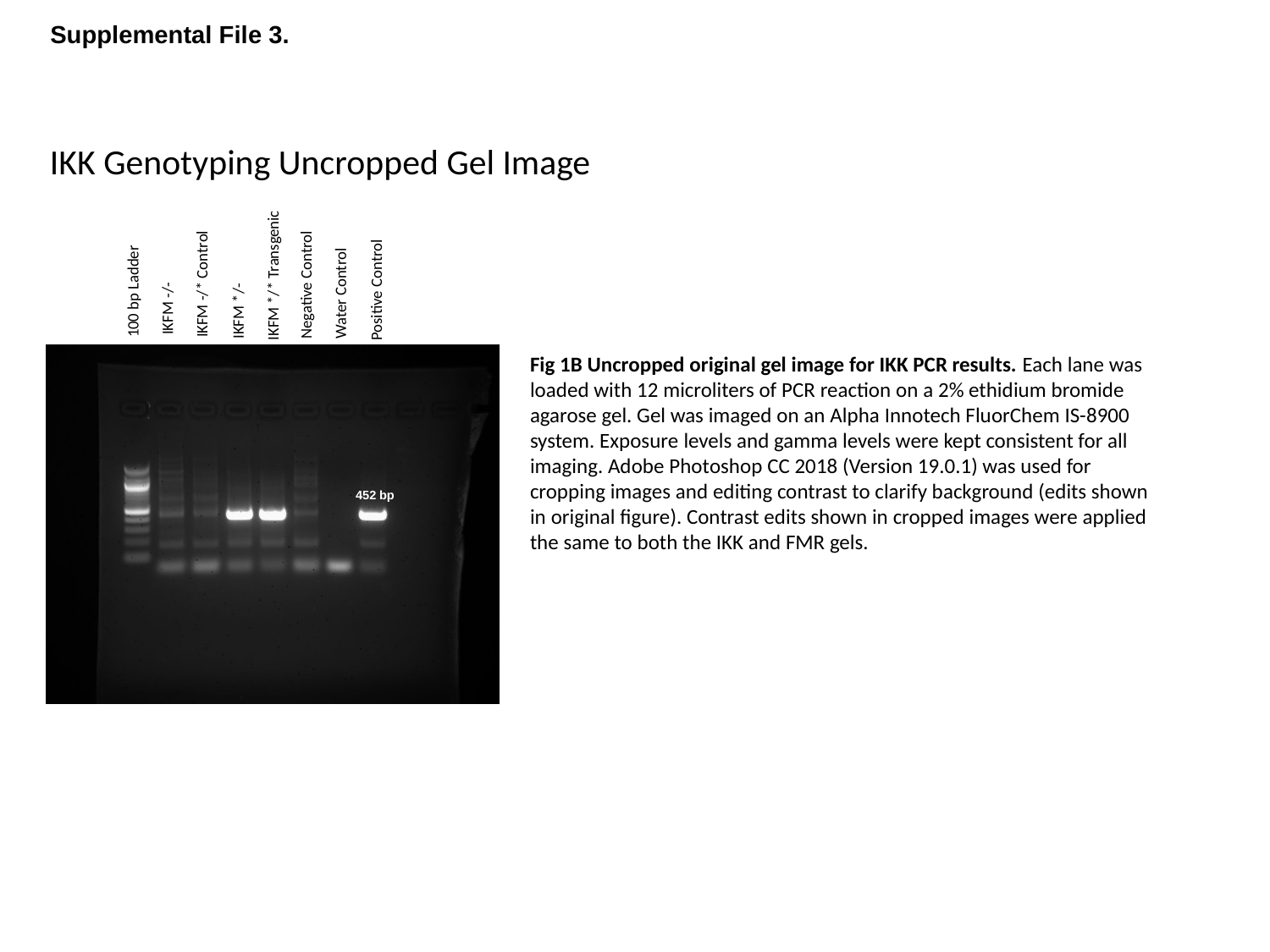

Supplemental File 3.
# IKK Genotyping Uncropped Gel Image
IKFM -/-
IKFM -/* Control
100 bp Ladder
IKFM */-
Negative Control
Water Control
Positive Control
IKFM */* Transgenic
Fig 1B Uncropped original gel image for IKK PCR results. Each lane was loaded with 12 microliters of PCR reaction on a 2% ethidium bromide agarose gel. Gel was imaged on an Alpha Innotech FluorChem IS-8900 system. Exposure levels and gamma levels were kept consistent for all imaging. Adobe Photoshop CC 2018 (Version 19.0.1) was used for cropping images and editing contrast to clarify background (edits shown in original figure). Contrast edits shown in cropped images were applied the same to both the IKK and FMR gels.
452 bp

## Slide 2
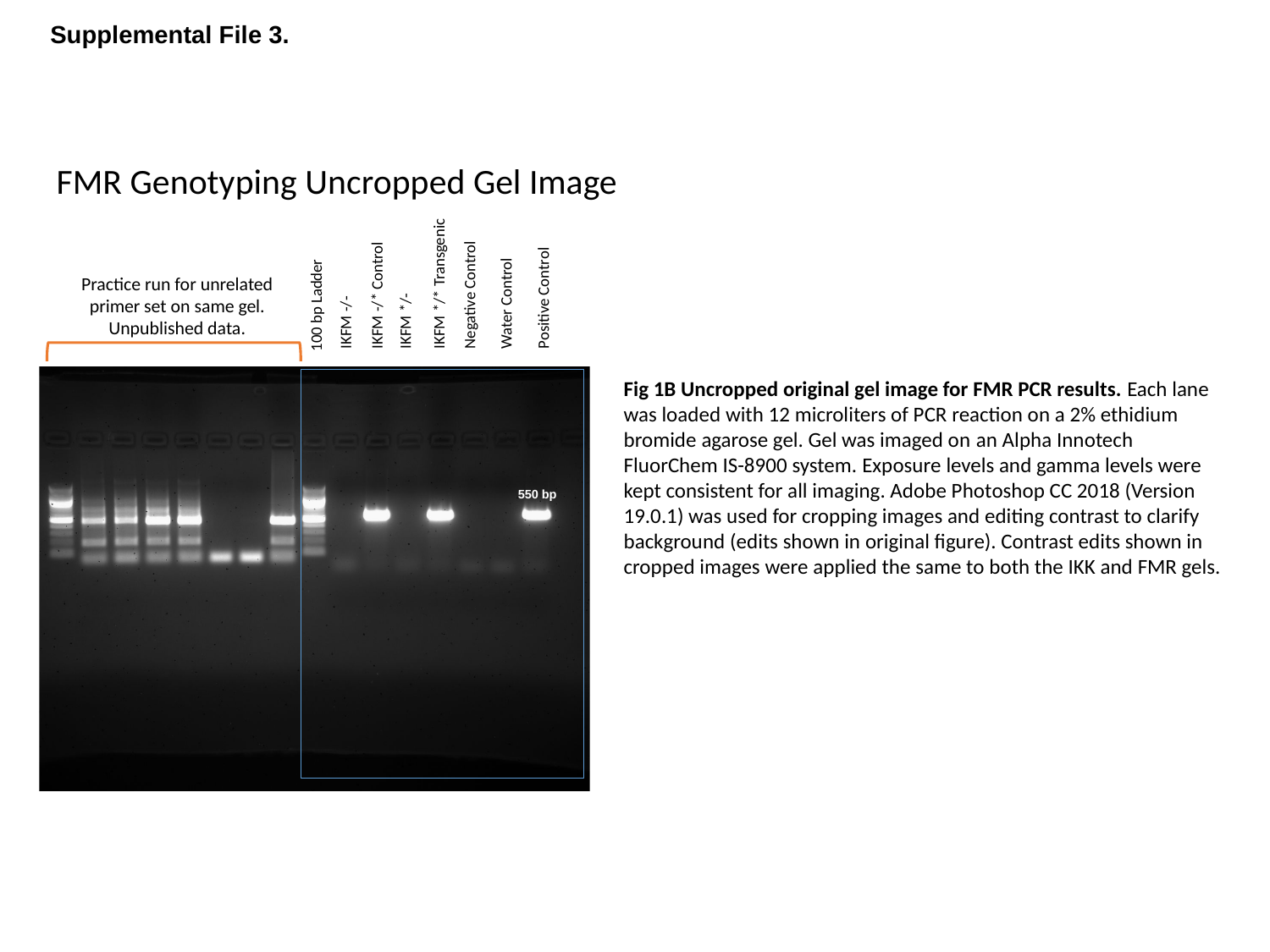

Supplemental File 3.
FMR Genotyping Uncropped Gel Image
IKFM -/-
IKFM -/* Control
IKFM */-
IKFM */* Transgenic
Negative Control
Water Control
Positive Control
100 bp Ladder
Practice run for unrelated primer set on same gel. Unpublished data.
Fig 1B Uncropped original gel image for FMR PCR results. Each lane was loaded with 12 microliters of PCR reaction on a 2% ethidium bromide agarose gel. Gel was imaged on an Alpha Innotech FluorChem IS-8900 system. Exposure levels and gamma levels were kept consistent for all imaging. Adobe Photoshop CC 2018 (Version 19.0.1) was used for cropping images and editing contrast to clarify background (edits shown in original figure). Contrast edits shown in cropped images were applied the same to both the IKK and FMR gels.
550 bp

## Slide 3
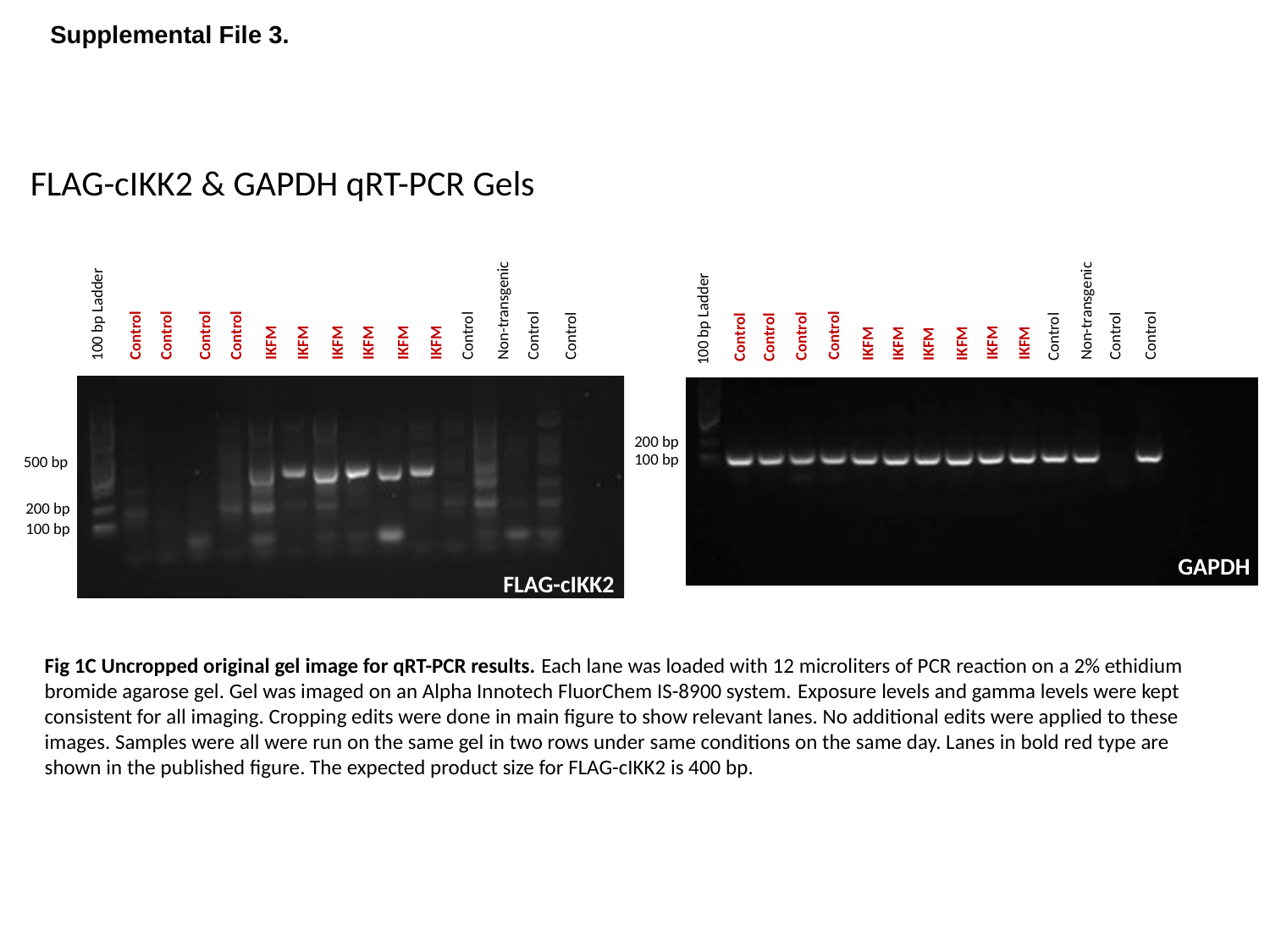

Supplemental File 3.
# FLAG-cIKK2 & GAPDH qRT-PCR Gels
100 bp Ladder
Control
Control
Control
Control
IKFM
IKFM
IKFM
IKFM
IKFM
IKFM
Control
Non-transgenic
Control
Control
100 bp Ladder
Control
Control
Control
Control
IKFM
IKFM
IKFM
IKFM
IKFM
IKFM
Control
Non-transgenic
Control
Control
200 bp
100 bp
500 bp
200 bp
100 bp
GAPDH
FLAG-cIKK2
Fig 1C Uncropped original gel image for qRT-PCR results. Each lane was loaded with 12 microliters of PCR reaction on a 2% ethidium bromide agarose gel. Gel was imaged on an Alpha Innotech FluorChem IS-8900 system. Exposure levels and gamma levels were kept consistent for all imaging. Cropping edits were done in main figure to show relevant lanes. No additional edits were applied to these images. Samples were all were run on the same gel in two rows under same conditions on the same day. Lanes in bold red type are shown in the published figure. The expected product size for FLAG-cIKK2 is 400 bp.

## Slide 4
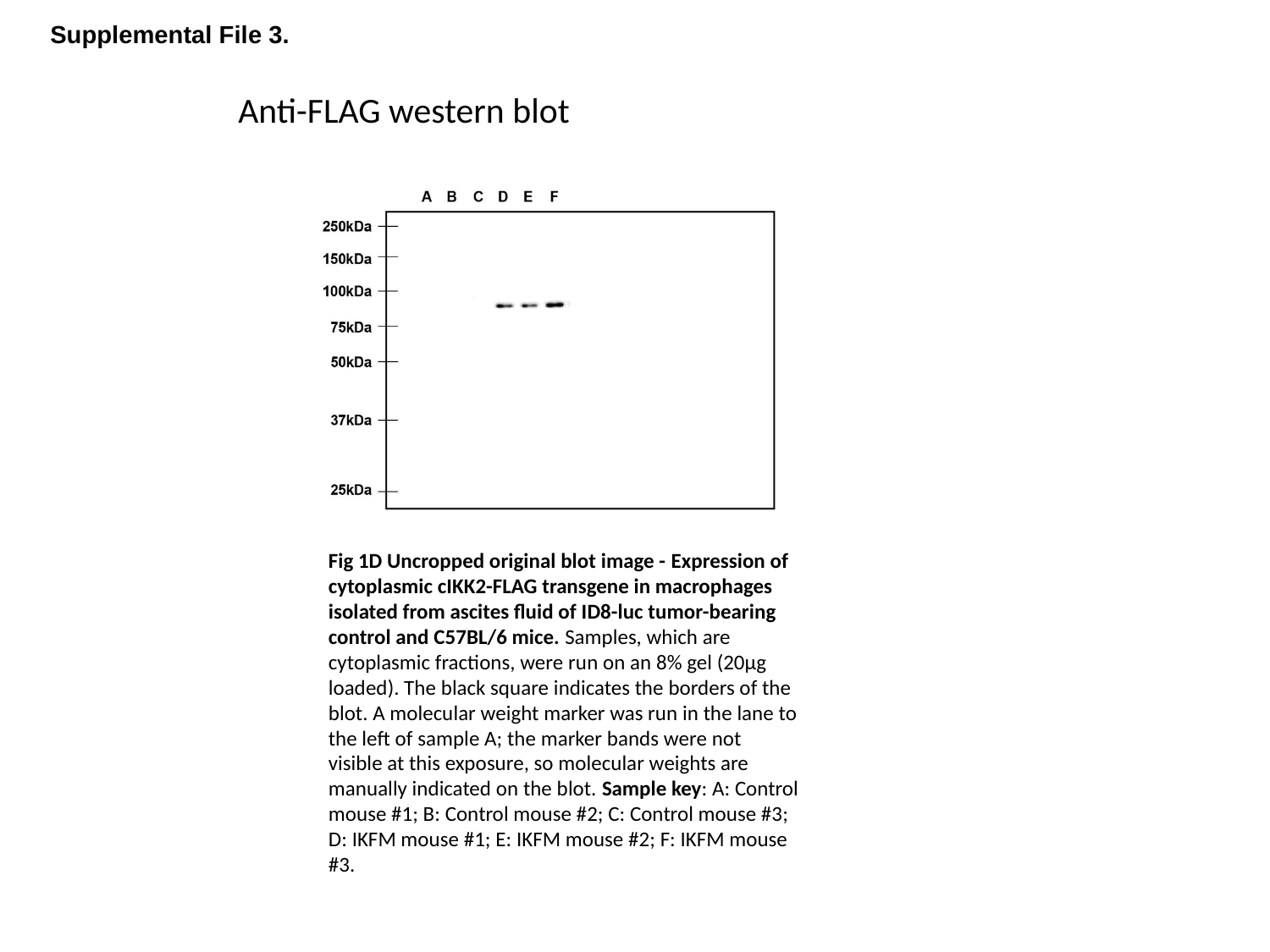

Supplemental File 3.
Anti-FLAG western blot
Fig 1D Uncropped original blot image - Expression of cytoplasmic cIKK2-FLAG transgene in macrophages isolated from ascites fluid of ID8-luc tumor-bearing control and C57BL/6 mice. Samples, which are cytoplasmic fractions, were run on an 8% gel (20µg loaded). The black square indicates the borders of the blot. A molecular weight marker was run in the lane to the left of sample A; the marker bands were not visible at this exposure, so molecular weights are manually indicated on the blot. Sample key: A: Control mouse #1; B: Control mouse #2; C: Control mouse #3; D: IKFM mouse #1; E: IKFM mouse #2; F: IKFM mouse #3.

## Slide 5
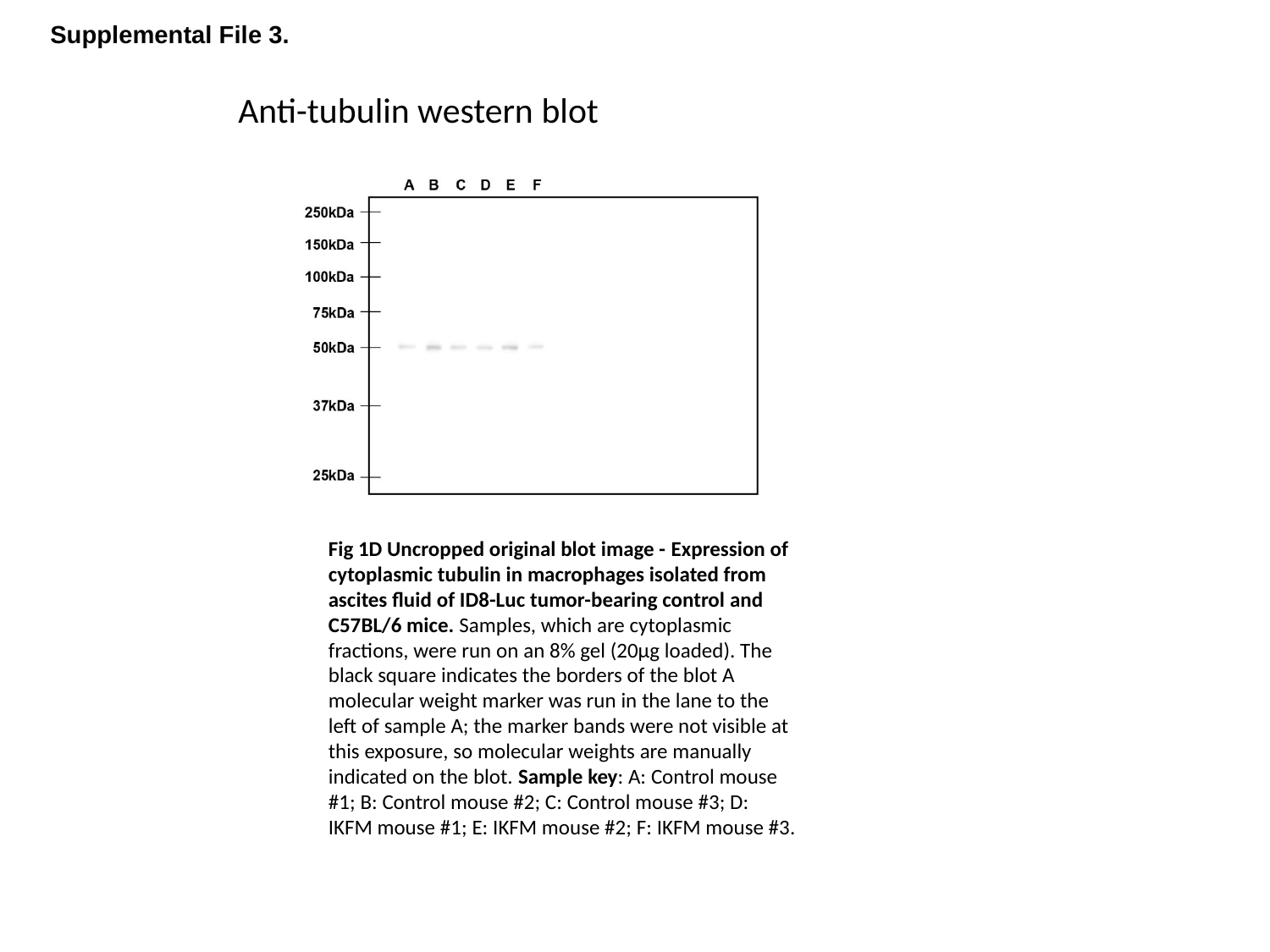

Supplemental File 3.
Anti-tubulin western blot
Fig 1D Uncropped original blot image - Expression of cytoplasmic tubulin in macrophages isolated from ascites fluid of ID8-Luc tumor-bearing control and C57BL/6 mice. Samples, which are cytoplasmic fractions, were run on an 8% gel (20µg loaded). The black square indicates the borders of the blot A molecular weight marker was run in the lane to the left of sample A; the marker bands were not visible at this exposure, so molecular weights are manually indicated on the blot. Sample key: A: Control mouse #1; B: Control mouse #2; C: Control mouse #3; D: IKFM mouse #1; E: IKFM mouse #2; F: IKFM mouse #3.

## Slide 6
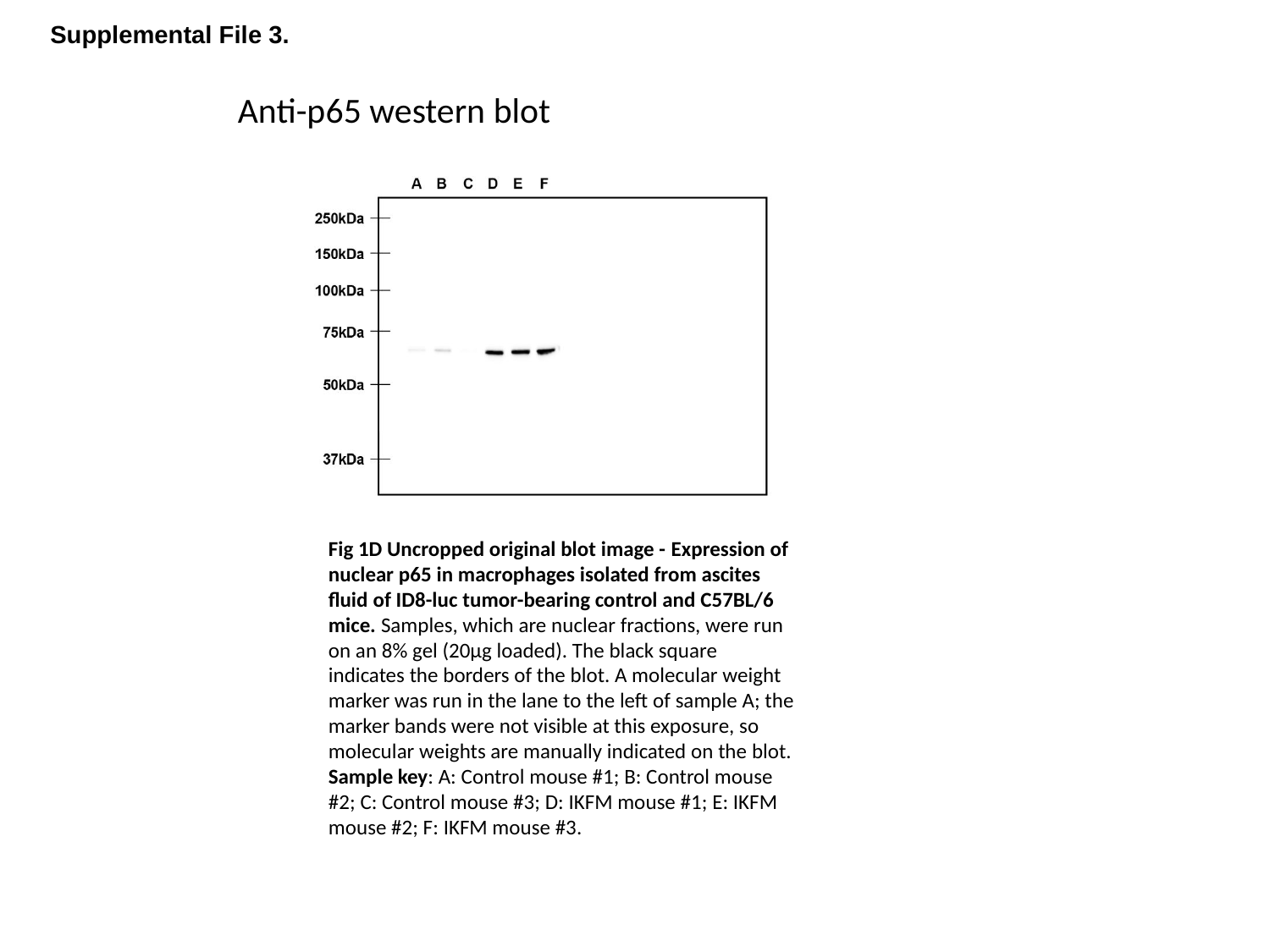

Supplemental File 3.
Anti-p65 western blot
Fig 1D Uncropped original blot image - Expression of nuclear p65 in macrophages isolated from ascites fluid of ID8-luc tumor-bearing control and C57BL/6 mice. Samples, which are nuclear fractions, were run on an 8% gel (20µg loaded). The black square indicates the borders of the blot. A molecular weight marker was run in the lane to the left of sample A; the marker bands were not visible at this exposure, so molecular weights are manually indicated on the blot. Sample key: A: Control mouse #1; B: Control mouse #2; C: Control mouse #3; D: IKFM mouse #1; E: IKFM mouse #2; F: IKFM mouse #3.

## Slide 7
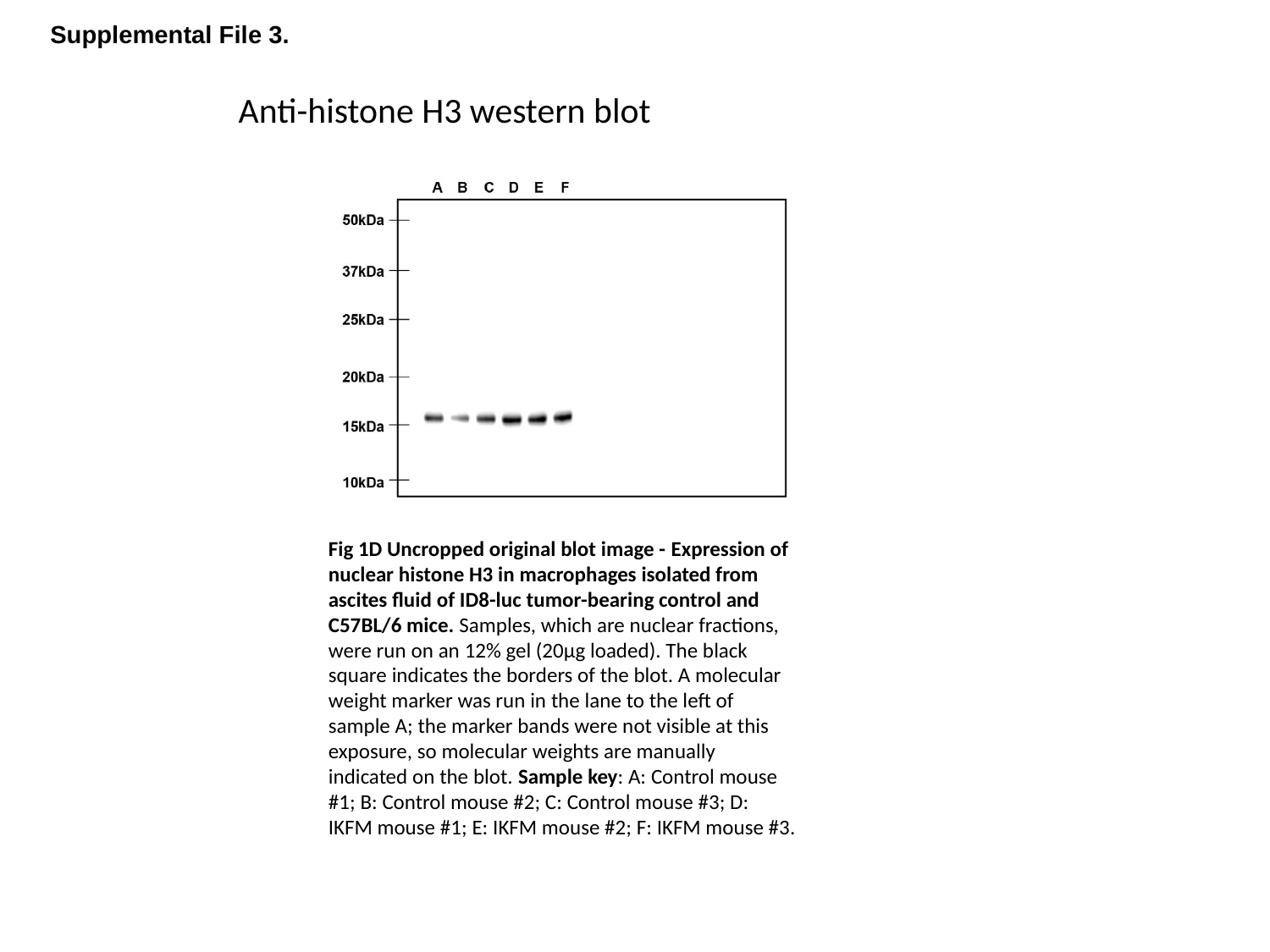

Supplemental File 3.
Anti-histone H3 western blot
Fig 1D Uncropped original blot image - Expression of nuclear histone H3 in macrophages isolated from ascites fluid of ID8-luc tumor-bearing control and C57BL/6 mice. Samples, which are nuclear fractions, were run on an 12% gel (20µg loaded). The black square indicates the borders of the blot. A molecular weight marker was run in the lane to the left of sample A; the marker bands were not visible at this exposure, so molecular weights are manually indicated on the blot. Sample key: A: Control mouse #1; B: Control mouse #2; C: Control mouse #3; D: IKFM mouse #1; E: IKFM mouse #2; F: IKFM mouse #3.
